# Evaluating the Epithelial-Mesenchymal Program in Human Breast Epithelial Cells Cultured in Soft Agar Using a Novel Macromolecule Extraction Protocol

**DOI:** 10.3390/cancers13040807

**Published:** 2021-02-15

**Authors:** Hiu Yeung Lau, Jingyi Tang, Patrick J. Casey, Mei Wang

**Affiliations:** 1 Programme in Cancer and Stem Cell Biology, Duke-NUS Medical School, National University of Singapore, Singapore 169857, Singapore; hiuyeung.lau@mohh.com.sg (H.Y.L.); jingyi.tang@u.duke.nus.edu (J.T.); patrick.casey@duke.edu (P.J.C.); 2 Department of Biochemistry, Yong Loo Lin School of Medicine, National University of Singapore, Singapore 117596, Singapore

**Keywords:** transformation, epithelial-mesenchymal transition, human mammary epithelial (HME) cells, soft agar colony formation assay, SV40 small T antigen, constitutively active RAS (CA-RAS)

## Abstract

**Simple Summary:**

Anchorage-independent soft agar colony formation assays have been widely used as an in vitro surrogate for in vivo tumour formation in xenograft studies, and has found much utility in studies such as cancer drug development. However, molecular characterisation of cells grown in soft agar has proven difficult and sometimes even impossible. We developed a set of new methods that allow DNA, RNA and proteins (including phosphoproteins) to be extracted from cells grown in soft agar, even without visible colony formation. We used these methods to demonstrate the role of the epithelial-mesenchymal program in the malignant transformation of a classical human mammary epithelial cell model.

**Abstract:**

The ability to grow in anchorage-independent conditions is an important feature of malignant cells, and it is well-established that cellular phenotypes in adherent cultures can differ widely from phenotypes observed in xenografts and anchorage-independent conditions. The anchorage-independent soft-agar colony formation assay has been widely used as a bridge between adherent cell cultures and animal tumor studies, providing a reliable in vitro tool to predict the tumorigenicity of cancer cells. However, this functional assay is limited in its utility for molecular mechanistic studies, as currently there is no reliable method that allows the extraction of biological macromolecules from cells embedded in soft-agar matrices, especially in experimental conditions where no visible colonies form. We developed a set of new methods that enable the extraction of DNA, RNA and proteins directly from cells embedded in soft agar, allowing for a wide range of molecular signaling analysis. Using the new methods and human mammary epithelial cells (HMECs), we studied the role of epithelial-mesenchymal transition (EMT) in the ability of HMECs to form colonies in soft agar. We found that, when cultured in soft agar instead of in adherent cultures, immortalized non-malignant HME-hTERT cells upregulated the epithelial program, which was noted to be necessary for their survival in this anchorage-independent condition. Overexpression of SV40 small T antigen (ST) or the EMT master-regulator SNAI1 negates this requirement and significantly enhances colony formation in soft agar driven by mutant-RAS. Interestingly, we found that, similar to SNAI1, ST also promotes EMT changes in HMECs, providing further support for EMT as a prerequisite for the efficient anchorage-independent colony formation driven by mutant-RAS in our HMEC model.

## 1. Introduction

The anchorage-independent soft-agar colony formation assay (hereafter referred to as CFA) has been an important tool for cancer research [1,2]. CFA has been widely used as a semi-quantitative assay to study various manipulations, either pharmacologic or genetic, on tumorigenesis in in vitro three-dimensional systems [3,4,5,6,7,8]. In CFAs, cells derived from adherent cultures or tumor samples are embedded in a three-dimensional matrix, usually comprised of a low concentration of agarose and cell culture media, and cultured according to individual experimental requirements. CFA has been considered to be one of the most stringent in vitro tests for transformation [2,9], as only malignant cells would have the ability to proliferate under the anchorage-independent condition. As such, the ability of cancer cells to form colonies correlates closely with that of in vivo tumorigenicity as measured by the formation of tumors in animal models [10,11,12].

Current mechanistic studies on cancer molecular signaling and the impact of anticancer therapies on these signaling processes depend heavily on cells cultured under adherent conditions. The advantages of such cultures, in contrast to that in soft agar, include easier manipulation of the cell growth milieu and straight-forward cell lysate preparation for the study of molecular and cellular characteristics. However, numerous studies have shown that growth and proliferation under adherent conditions are not limited to cancer cells; many non-malignant cells proliferate well under these conditions. Therefore, observations made under these conditions may not be relevant in settings outside adherent cultures. For example, it has been observed that a cancer cell line and a non-malignant cell line may demonstrate similar sensitivity/resistance to an anti-cancer agent when assayed under adherent culture conditions. Whereas in CFAs, the cancer cells may be much more sensitive to the drug treatment, i.e., IC_50_ values derived from adherent cell viability assays may differ significantly from those obtained through the analysis of soft-agar CFAs [5,13]. The differences between cells grown in these two culture conditions also manifest in gene expression and protein function studies [13]. Therefore, adherent cultures may not always be the most relevant approach in studying cancer cell growth, proliferation and the associated molecular pathogenesis.

Researchers also face challenging issues with regards to in vivo tumorigenesis assays such as tumor xenografts in mice. Although there are obvious advantages associated with in vivo xenograft assays, these experiments tend to be much longer and costly. Moreover, any downstream signaling analysis is only possible if the transplanted cells actually form tumors, making studies that involve manipulating cell signaling with the aim of inhibiting tumor formation virtually impossible.

While many have recognized the advantages of using CFAs to evaluate tumorigenesis, molecular mechanistic studies on the cancer cells grown in such conditions are limited due to the difficulties associated with isolating cells and purifying cellular macromolecules, such as genomic DNA, RNA and protein, from the agarose matrix. There have been recent efforts to isolate single colonies for downstream analysis [14,15]; however, these approaches have major limitations as, similar to xenograft studies, they cannot be used to evaluate signaling program alterations that significantly reduce tumorigenesis and therefore colony formation. Other limitations of such approaches include the scarce amount of macromolecules available in single colonies and the inherent selection bias introduced by the process of “picking” of single colonies.

Given that the CFA is a more relevant in vitro assay for studying tumorigenicity compared to adherent culture, it is important to develop reliable methods to isolate macromolecules from cells in soft agar to enable direct downstream mechanistic analyses. However, there is no method currently available that allows for the convenient extraction of reasonable quantities of high-quality macromolecules directly from the population of cells embedded in the agarose matrix for biologically relevant molecular evaluations. The development of such a method would greatly facilitate analyses of studies with CFA cultures using platforms such as quantitative PCR (q-PCR), next-generation sequencing and protein quantification and characterization assays.

Oncogenes and tumor suppressors have been sequentially introduced and removed, respectively, into human mammary epithelial (HME) cells to evaluate the effects of various factors on the acquisition of malignant properties [16,17]. In a classic model, human telomerase (hTERT) was first introduced into HME cells to generate the immortalized HME-hTERT cells [17,18]. While HME-hTERT cells are “immortal” when grown in an appropriate medium under adherent conditions, they lack the capacity of surviving and proliferating in anchorage-independent in vitro growth conditions, such as in soft agar, or forming tumors in xenograft models. Further, numerous studies have shown that HME-hTERT cells are not in a ready state to be induced by RAS oncogenes to grow in suspension or form tumors [17,19]. In many HMEC studies, SV40 small T antigen (ST) oncogene is often introduced to the immortalized HME-hTERT cells to generate a “transitional state” cell line, namely the HME-ST, which, although still could not form colonies in soft agar on its own, have now acquired the ability to be made fully malignant by oncogenes such as mutant-RAS, especially in combination with p53 knockdown. CFAs have also been used extensively, as a surrogate for in vivo tumor formation studies, to measure the tumorigenic potential of HME cells upon manipulation with different oncogenic factors and signaling programs [10,17,20,21,22].

Epithelial-mesenchymal transition (EMT) has well-known roles in normal physiological processes such as embryonic development and wound healing [23]. In recent years, EMT has garnered much attention in cancer research, as the transition to a mesenchymal state is associated with increased cancer progression and metastatic potential, as well as the acquisition of so-called cancer stemness [23,24,25]. In the HME model, EMT promotes stem-like properties and increases tumorigenic potential [22,26]. EMT-promoting growth factors and transcription factors such as TGF-β, ZEB1, ZEB2, TWIST1 and SNAIL1/2 work together with oncogenes such as RAS and ERBB-2 to achieve enhanced tumorigenicity [21,22]. Interestingly, although it has been shown that EMT transcription factors are able to work together with oncogenic RAS in HME cells to achieve efficient transformation [21], it has never been reported whether the manipulations preceding the introduction of oncogenic RAS would lead to EMT. These manipulations, such as the introduction of ST antigen, neither directly transform nor increase the proportion of the stem-like cells in HME cells [17,22,26]. Instead, they appear to create a transitional state that positions HME cells for a strong transformation phenotype when oncogenic RAS is expressed. However, it is not known if EMT plays a role in the formation of this transitional state and whether EMT is essential in mutant-RAS-induced transformation of HME cells.

In order to investigate the critical role of EMT in different transitional states in HME cells, the cell signaling study needs to be performed in a setting that allows phenotypic changes to reflect genotypic changes. As the critical in vitro phenotypic difference between these populations of HME cells is that the so-called transformed HME cells have acquired the ability to grow in an anchorage-independent fashion, it is a prerequisite to develop a reliable method to isolate DNA, RNA and proteins from the cells grown in the soft-agar matrix. Taking reference from reports on the extraction of RNA from polysaccharide-rich plant tissues and matrices [27,28,29], we made modifications and developed a method to extract high-quality RNA and genomic DNA from cells embedded in the soft agar. We also developed a method to extract proteins from cells embedded in soft agar by modifying the methanol-chloroform precipitation procedure [30,31]. Applying these methods, we are able to gain insights into the molecular changes between the different transformation states of HME cells that were not possible by studying the adherent culture lysates alone.

## 2. Materials and Methods

### 2.1. Adherent Cell Culture and Studies

**Figure 1:**
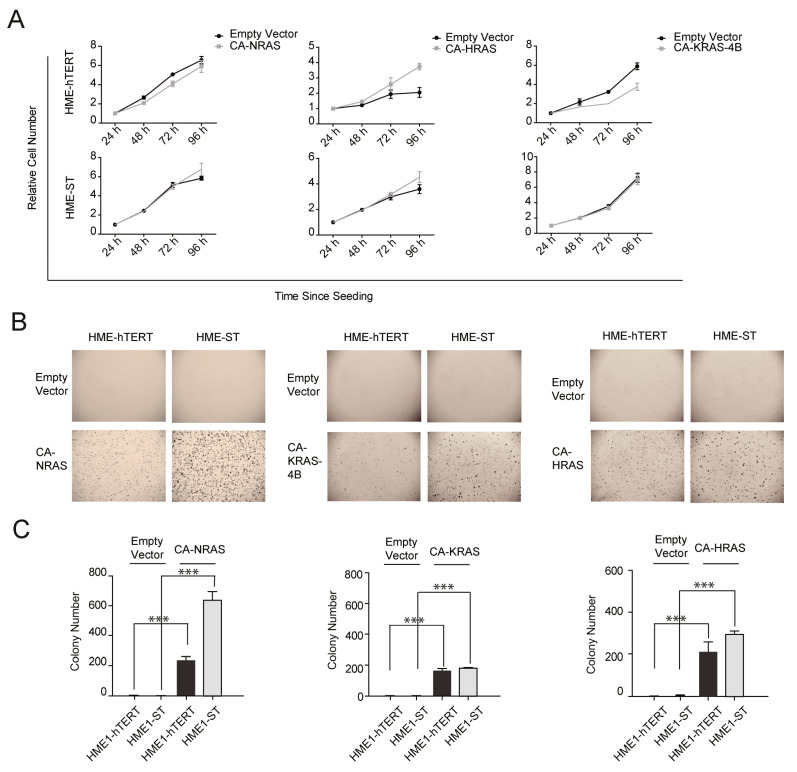
The proliferation phenotypes of various human mammary epithelial cells in adherent culture or in anchorage-independent soft-agar growth condition. (**A**) Proliferation of human mammary epithelial (HME) cells at different stages of transformation cultured under adherent condition. HME-hTERT: immortalized human mammary epithelial cells; HME-ST: HME-hTERT also expressing SV40 small T antigen. Each isoform of constitutively active RAS was introduced into the two cell lines; grey lines and black lines represent the cells with or without the expression of the indicated isoforms of constitutively active RAS: CA-NRAS, CA-HRAS and CA-KRAS. (**B**) Representative microscopic images documenting colony formation of the indicated HME cells, as in panel A, cultured in soft agar for 11 days. (**C**) Quantitation of the data from (**B**), summed from three representative microscopic fields for each condition. Data are presented as mean ± SD; *** *p* ≤ 0.001 (Student’s *t*-test). All studies presented above have been repeated twice with similar results.

HME-hTERT cells were obtained from ATCC, with the subsequent stable expression of SV40 small T antigen (HME-ST), and further, an shRNA against p53 (HME-shp53), performed as described in our previous study [10] (Figure 1A). Different isoforms of constitutively active RAS (CA-RAS) were introduced into HME-hTERT, HME-ST and HME-shp53 cells, respectively, using the retroviral expression vector pBABE-puro; the empty vector was used as a negative control. All HME-derived cells were maintained in MEGM™ BulletKit™ Mammary Epithelial Cell Growth Medium (Lonza, Basel, Switzerland). Mia-PaCa-2 cells were obtained from ATCC and cultured according to guidelines. For viability studies, cells were seeded in 96-well tissue culture plates and cultured under specified conditions for each study for 24, 48, 72 and 96 h, followed by a tetrazolium-based cell viability assay (CellTiter 96^®^ AQueous One Solution Cell Proliferation Assay, Promega, Madison, WI, USA) to quantify live cells according to the manufacturer’s protocol. Relative cell viability values were determined with respect to the readings obtained at the time of seeding. For comparative expression analysis, HME cells were cultured in DMEM medium supplemented with 10% FBS, 0.5 μg/mL hydrocortisone (Sigma Aldrich, St. Louis, MI, USA), and 5 μg/mL insulin (I-DNA Biotech, Singapore, Singapore) for 48 h using standard tissue culture conditions. Cells were subsequently harvested according to the downstream assay to be performed.

### 2.2. Anchorage-Independent Soft-Agar Colony Formation Assay

To evaluate the soft-agar colony-forming ability of HME cells, Dulbecco’s Modified Eagle’s Medium (DMEM, Nacalai Tesque, Kyoto, Japan) supplemented with 10% Fetal Bovine Serum (FBS, GE Healthcare, Chicago, IL, USA) (*v/v*) and penicillin (100 U/mL)/streptomycin (100 μg/mL) (GIBCO/Thermo Fisher, Waltham, Massachusetts, United States) was used (1× DMEM). Specifically, a layer of 0.5% noble agar (Sigma Aldrich) in DMEM + 10% FBS (diluted from 2× DMEM + 20% FBS) was first laid in tissue culture plates and allowed to solidify at room temperature as the “base agar”. Subsequently, HME cells of interest were mixed with 0.25% noble agar in DMEM + 10% FBS as the “cell agar” laid on top of the “base agar” layer. Once the “cell agar” solidified, the top medium constituted of DMEM, 10% FBS, 0.5 μg/mL hydrocortisone and 5 μg/mL insulin was added on top. The plates were cultured under standard tissue incubation conditions. The media on top was refreshed every week of culturing. The colonies are visualized, usually after about 2 weeks of culturing, by normal light microscopy or by staining with methylthiazolyldiphenyl-tetrazolium bromide (MTT) (Sigma Aldrich) per manufacturer’s protocol. The colonies were photographed using an Olympus SZX16^®^ Research Stereo Microscope with a 3.2× objective. For the purpose of extraction of DNA, RNA and proteins from soft agar cultures, a similar protocol was employed with the following modifications: low melting point (LMP) agarose (Invitrogen/Thermo Fisher, Waltham, MA, USA) was used instead of noble agar; the “base agar” consisted of 0.65% LMP agar in DMEM and 10% FBS, and the “cell agar” consisted of HME cells of interest in 0.325% LMP agar in the same medium as the base layer; all solidification steps were done at 4 °C for 15 min, and the same “top media” were used for culturing. Cultures were harvested after 3 days for sample preparation.

To visualize the apoptotic cells in the soft agar, a modified propidium iodide (PI) staining method was used. At the time of assay, the top medium was changed to that containing 10 µL/mL propidium iodide (PI; Sigma Aldrich) and the assay incubated in the dark at 37 °C for 30 min. The apoptotic cells, which stain positive for PI, were visualized and photographed using Olympus SZX16^®^ Research Stereo Microscope with a 3.2× objective. The resultant images were analyzed using ImageJ software (NIH).

### 2.3. RNA and Genomic DNA Extraction from Cells Embedded in Soft Agar

The extraction buffer (termed CTAC buffer), consisting of 2% *w/v* cetyltrimethylammonium chloride (CTAC, Sigma Aldrich), 2% *w/v* polyvinylpyrrolidone (PVP-40, Sigma Aldrich), 2 M sodium chloride, 100 mM Tris-HCl at pH 8.0 and 20 mM ethylenediaminetetraacetic acid (EDTA, Sigma Aldrich) in RNase-free water, was autoclaved, and 2% *v/v* beta-mercaptoethanol (Sigma Aldrich) was added immediately prior to use. The “top media” were first removed from the soft-agar CFA, and the pre-warmed CTAC buffer (65 °C) was added to the soft agar (5:1 to volume CTAC to soft-agar); the mixture of buffer with both the base and cell agar was vigorously mixed and vortexed. An equal volume of chloroform (Sigma Aldrich) was added to the homogenate, mixed vigorously and collected in centrifuge tubes. The mixture was centrifuged at 15,000× *g* for 5 min at room temperature, and the resultant clear upper phase was collected in new centrifuge tubes. An equal volume of isopropanol (Sigma Aldrich) was then added to the collected supernatant, and the mixture was centrifuged at 15,000× *g* for 15 min at room temperature. The pellet was washed with 70% ethanol and collected by centrifugation at 15,000× *g* for 5 min; the pellet was air-dried. For RNA extraction, the pellet was resuspended in RNase-free water and incubated with DNase I (Fermentas/Thermo Fisher, Waltham, MA, USA) at 37 °C for 30 min to remove any DNA. Subsequently, RNA was purified and collected using the RNeasy^®^ Mini Kit (Qiagen, Venlo, The Netherlands) as per protocol. The column was spin at 8000× *g* for 30 s, then washed 4 times with 500 µL of RPE buffer (Qiagen). RNA was eluted from the column using an appropriate amount of RNase-free water. For genomic DNA (gDNA) extraction, the air-dried nucleic acid pellet was resuspended in water and incubated with RNase A (Qiagen) at 37 °C for 30 min to remove any RNA. Subsequently, DNA was purified using the DNeasy^®^ Blood and Tissue Kit (Qiagen) following the manufacturer’s protocol.

### 2.4. Protein Extraction from Cells Embedded in Soft Agar

For extracting proteins from cells grown in CFA, the top media were first removed as thoroughly as possible. Phosphate-buffered saline (PBS) was added to mix with agar in the well; the solution and the agar were then vigorously mixed and placed in a 15 mL centrifuge tube with an additional 10 mL of PBS for washing. The mixture was centrifuged at 700× *g* for 2 min and the supernatant discarded. The process of mixing the agar with fresh PBS wash and centrifugation was repeated twice. The resultant pellet was transferred into microcentrifuge tubes and snap-frozen with liquid nitrogen. The samples can be processed immediately or stored at −80 °C until ready for protein extraction. To begin protein extraction, RIPA buffer (Thermo Fisher, Waltham, MA, USA) containing protease and phosphatase inhibitors (Roche, Basel, Switzerland) and β-mercaptoethanol at a concentration of 2% were added to the agar pellet; the mixture was heated for 10 min at 95 °C and immediately chilled and incubated on ice for 1 h, followed by centrifugation at 15,000× *g* for 20 min at 4 °C. The supernatant was transferred to a new microcentrifuge tube, mixed thoroughly with an equal volume of 100% methanol and 1/4 volume of chloroform. This mixture was centrifuged at 15,000× *g* for 3 min, whereupon the upper phase was discarded, and an equal volume of 100% methanol was thoroughly mixed with the interface and lower phase. The mixture was again centrifuged for 3 min at 15,000× *g*; the resultant pellet was air-dried and resuspended in an appropriate amount of RIPA buffer + protease/phosphatase inhibitors for subsequent analysis.

### 2.5. Assessment of RNA Quality

RNA quality was assessed using a bleach-supplemented agarose electrophoresis protocol [32]. Briefly, biotechnology grade agarose (1st Base, Singapore, Singapore) (to achieve 1% *w*/*v*) in 1% *v*/*v* household bleach containing 6% sodium hypochlorite (Clorox^®^) and 1× Tris-Acetate-EDTA (TAE) buffer (1st Base) was heated to boil for 2 min. Ethidium bromide was then added to the mixture and the bleach-agarose gel allowed to solidify. The isolated RNA samples were loaded onto the gel and subjected to standard electrophoresis in fresh TAE buffer at 100–120 V constant voltage. For advanced RNA integrity analysis, the Agilent Technologies 2100 Bioanalyzer was used to determine the RNA Integrity Number (RIN) of individual samples.

### 2.6. Commonly Used Molecular Techniques

Quantitative real-time polymerase chain reaction analysis, immunoblot analysis for protein expression and small-hairpin RNA (shRNA) expression by transduction of lentiviral expression constructs are reported in our previous publications and are briefly described as follows [33]. From two-dimensional cultures, RNA was obtained with the RNeasy^®^ Mini Kit (Qiagen) or the Tissue Total RNA Mini Kit (FATRK 001-2, Favorgen Biotech Corporation, Ping-Tung, Taiwan) as per protocol. From RNA (either from two- or three-dimensional cultures), cDNA was obtained using the ReverTra Ace qPCR RT Master Mix (FSQ-201, Toyobo, Osaka, Japan). Quantitative real-time PCR was performed using Thunderbird SYBR qPCR Mix (Toyobo) with an Applied Biosystems 7900HT RT-PCR system. Data analysis was subsequently performed using the comparative Ct method, and the relative expression values of each gene in specific experimental conditions are presented as (2^−ΔCt^ × 10^4^). Primers used for q-RT-PCR are listed in the Supplementary Materials Table S1. Standard sodium dodecyl sulfate–polyacrylamide gel electrophoresis (SDS-PAGE) methods were employed on cell lysates either obtained from three-dimensional cultures as described above, or from cells in two-dimensional adherent cultures lysed using RIPA buffer containing protease and phosphatase inhibitors. Immunoblots were then performed using the following antibodies: (from Cell Signaling Technology, Danvers, MA, USA) Caspase 3 (#9662), p21^Cip1^ (#2947S), Cyclin D1 (#2926S), E-cadherin (#3195) and Snail (#3879); (from Abgent, San Diego, CA, USA) Beta-actin (AM1829B); (from BD Transduction Laboratories, Franklin Lakes, NJ, USA) N-Cadherin (610920); (from Thermo Fischer) pan-Ras (MA1-012); and (from Santa Cruz Biotechnology, Dallas, TX, USA) Occludin (H-279) (sc-5562) and Desmoplakin (sc-33555). Knockdown of SNAI1 and CDH1 were performed using the third-generation lentiviral shRNA vector pLL3.7, with shRNA sequences listed in Table S1. Original western blots are listed in Figure S4.

## 3. Results

### 3.1. Different States of Transformation of HME Cells Can Only Be Reliably Evaluated by the Changes in the Proliferative Ability under Anchorage-Independent Growth Conditions, but Not in Adherent Culture

It is not a new notion that the proliferation rate of a cell line in adherent culture does not correlate well with its state of transformation nor ability to form colonies in soft agar. To study the behavior of HMECs cultured under these two conditions, we compared the proliferation of HME-hTERT and HME-ST (HME-hTERT cells stably expressing SV40 small T antigen) cells, with or without the expression of the G12V-activating mutant of different isoforms of RAS, under either adherent conditions or with soft-agar CFAs. Under adherent conditions, all cell lines proliferate albeit some do so at different rates (Figure 1A). Notably, expression of the RAS-G12V mutant proteins (herein referred to as constitutively active or CA-RAS) had no consistent impact on the proliferation rates on HME-hTERT and HME-ST cell lines in adherent cultures (Figure 1A), despite the known tumorigenic function of mutant-RAS. In contrast, soft-agar colony formation assays (CFAs) revealed substantial differences in the proliferation abilities between control and CA-RAS-expressing HME cells; neither HME-hTERT nor HME-ST cells form colonies when cultured in CFAs without oncogenic RAS (Figure 1B,C). Furthermore, even though the expression of CA-RAS results in visible colony formation in both HME-hTERT and HME-ST cells, HME-ST cells form significantly more conspicuous colonies, consistent with the notion that ST expression significantly enhances the susceptibility of HME cells to mutant-RAS-induced proliferation under the anchorage-independent growth condition (Figure 1B,C). These differences are not due to the levels of mutant-RAS expression (Supplementary Materials Figure S1), suggesting that the enhanced proliferative ability in soft agar is brought about by the expression of ST and the consequent signaling changes.

### 3.2. The Transition from Adherent to Anchorage-Independent Culture Is Accompanied by Significant Upregulation of Epithelial Signatures in the Immortalized, but Non-Malignant, HME-hTERT Cells

Given the stark contrast between the proliferation of the various HME cells under adherent and soft-agar culturing conditions, we sought to investigate the differences in molecular signatures, such as gene expression and protein markers, between the cells grown under the two conditions. To this end, we had to develop a method of extracting RNA and proteins from the cells embedded in soft agar. For the purpose of extracting high-quality RNA from cells embedded in soft agar, we modified a plant tissue RNA extraction method [27,28,29]; for the purpose of extracting proteins, we modified the methanol-chloroform precipitation procedure [30,31]. Using these methods, which are detailed in Materials and Methods and illustrated in Supplementary Materials (Figure S2A–F), we showed that we can obtain DNA, RNA and proteins (including phosphoproteins) of comparable quality as those extracted from cells grown in adherent culture (see Supplementary Materials (Figure S2A–F) and accompanying figure legend). The major advantage of this method is that it allows the extraction of these macromolecules from cells embedded in soft agar even when there is no colony formation, a superior feature over xenograft studies and single-colony isolation protocols.

**Figure 2:**
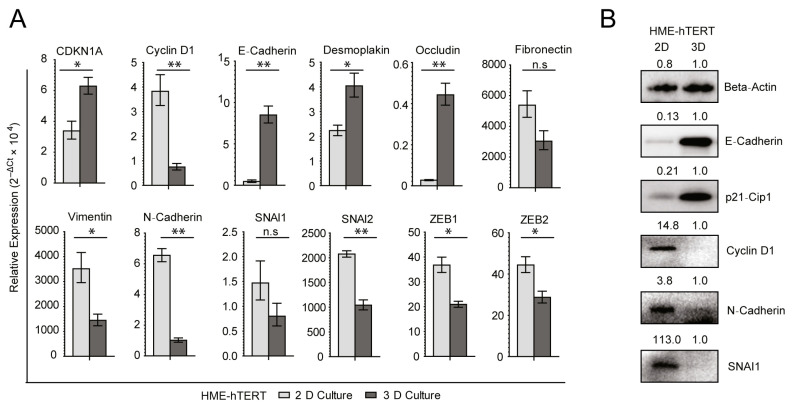
The non-malignant but immortal HME-hTERT cells upregulate the expression of genes associated with epithelial differentiation and cell cycle arrest when transferred from adherent to anchorage-independent soft-agar culture. (**A**) Q-RT-PCR analysis of RNA samples obtained from adherent (2-D culture, light grey bar) and soft-agar (3-D culture, dark grey bar) cultures of HME-hTERT cells of the indicated genes. Statistical significance is reported as p values computed using mean −ΔCt values and normalized SD; ** *p* ≤ 0.01; * *p* ≤ 0.05; n.s: Not Significant. Data shown are the relative expressions [(2^−ΔCt^ × 10^4^) ± normalized SD] from duplicate determinations of a single experiment that is representative of three such experiments. (**B**) Immunoblot images and densitometry band intensity analysis of the indicated proteins extracted from HME-hTERT cells cultured in adherent (2D) and soft-agar (3D) conditions. Beta-actin was used as the loading control. The data are representative of three biological repeated experiments.

Applying the new methods, we studied the differences in gene expression between HME-hTERT cells growing in adherent cultures and embedded in soft agar. We found that the CDK inhibitor CDKN1A (p21^Cip1^) was upregulated and CCND1 (cyclin D1) was downregulated when HME-hTERT cells are cultured in soft agar compared to adherent culture, which indicates that they went into cell cycle arrest when placed in an anchorage-independent growth condition (Figure 2A). This finding is consistent with the fact that non-malignant cells do not proliferate to form colonies in soft agar. More strikingly, the anchorage-independent condition led to the upregulation of epithelial markers such as E-cadherin (CDH1), occludin (OCLN) and desmoplakin (DSP) in HME-hTERT cells (Figure 2A). Consistent with this upregulation of the epithelial program, expression of mesenchyme-associated genes such as vimentin (VIM), N-cadherin (CDH2), SNAI2, ZEB1 and ZEB2 was significantly downregulated, with fibronectin (FN1) and SNAI1 showing a trend toward downregulation although statistical significance was not achieved (Figure 2A). The changes in gene expression are corroborated by protein levels, which showed corresponding changes of E-cadherin, N-cadherin, p21^Cip1^ and cyclin D1 protein levels when HME-hTERT cells were cultured in soft agar compared to adherent conditions (Figure 2B). SNAI1 protein levels are also clearly downregulated in soft-agar culture (Figure 2B). These RNA and protein expression signatures demonstrate that the HME-hTERT cells become more epithelial-like and undergo cell cycle arrest when placed in anchorage-independent culture, consistent with the observation that these non-malignant cells, unlike their malignant counterparts, cannot form colonies efficiently even when a strong proliferation signal such as mutant-RAS isoforms is expressed.

### 3.3. CDH1/E-Cadherin Is Required for the Survival and CA-RAS-Stimulated Proliferation of HME-hTERT Cells Embedded in Soft Agar

**Figure 3:**
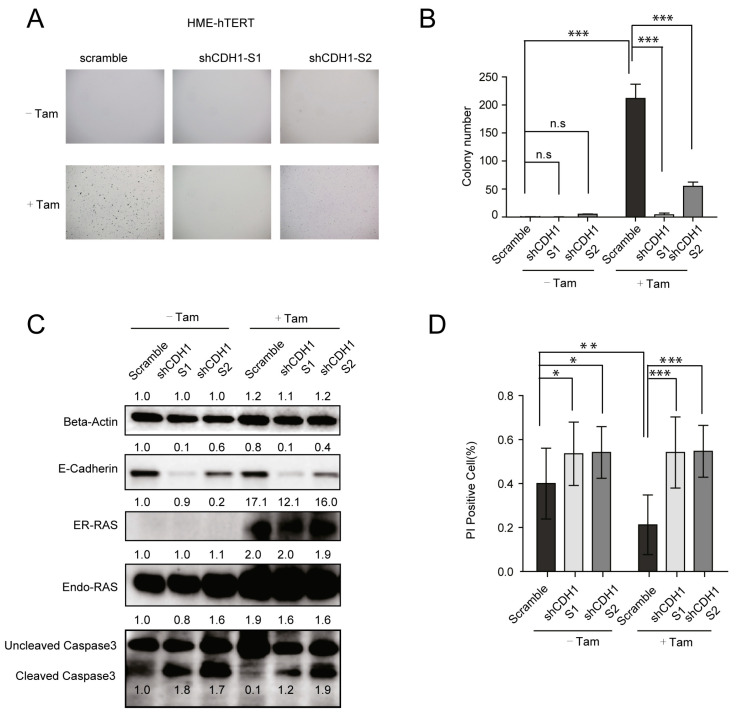
The upregulation of E-cadherin (CDH1) in HME-hTERT is necessary for cell survival and oncogenic RAS-stimulated proliferation under the soft-agar growth conditions. (**A**) Representative microscopic images of soft-agar colonies of HME-hTERT cells expressing ER-tagged CA-NRAS inducible by tamoxifen and expressing either a control shRNA (scramble) or two different shRNAs targeting CDH1. (**B**) Quantitation of the data from the experiment in panel A. The data represent the mean and SD of colony counts from five representative microscopic fields from the indicated condition. The study was repeated 3 times with similar results. *** *p* ≤ 0.001 (Student’s *t*-test). (**C**) Immunoblot analysis of protein samples extracted from HME-hTERT cells cultured in soft agar; the cells were treated the same way as in panels A and B in which expression ER-tagged mutant-NRAS was induced by tamoxifen (Tam) in the presence and absence of CDH1 knockdown. (**D**) Apoptosis analysis of HME-hTERT cells cultured in soft agar. The cells were seeded under the same experimental conditions as in panel A; apoptotic cells were quantitated by propidium iodide (PI) staining as detailed in Materials and Methods. Data shown represent the mean percentage of the PI-positive HME-hTERT cells in soft-agar. Quantification was done using five representative microscopic fields from a single experiment that has been repeated with similar results. The data are presented as mean ± SD. * *p* ≤ 0.05, ** *p* ≤ 0.01, *** *p* ≤ 0.001 , n.s: Not Significant, (Student’s *t*-test).

Epithelial-mesenchymal transition (EMT) is a well-established process related to malignant transformation and tumor progression [21,22]. The loss of CDH1/E-cadherin, a key factor associated with epithelial differentiation and cell adhesion [34], is a hallmark of malignant progression for many cancers including breast cancer [35]. We observed that, under anchorage-independent conditions, the upregulation of CDH1 expression in HME-hTERT cells is correlated with the cell cycle arrest and absence of colony formation. Therefore, we proceeded to investigate whether downregulating CDH1 would result in enhanced colony formation in soft agar, with and without mutant-RAS expression in HME-hTERT cells. Two independent shRNAs targeting CDH1 were stably introduced into HME-hTERT cells, followed by a stable introduction of tamoxifen-inducible mutant-NRAS (ER-CA-NRAS). NRAS was used in the study because of its high efficiency in inducing colony formation in HMECs (Figure 1B,C). These cells were then placed in soft agar with and without tamoxifen to assess their capacity to form colonies. Contrary to our expectation, silencing CDH1 in HME-hTERT cells expressing ER-CA-NRAS resulted in the reduction of the number of colonies in comparison to cells without CDH1 knockdown (Figure 3A,B). Analysis of protein samples extracted from cells cultured in soft agar revealed that CDH1 downregulation led to increased cleavage of caspase 3, suggesting that loss of CDH1 triggers apoptosis in HME-hTERT cells cultured in soft agar (Figure 3C).

To further evaluate the role of CDH1 in the survival of HME-hTERT cells, we used in situ propidium iodide (PI) staining of cells embedded in soft agar to identify and quantify apoptotic cells. When the mutant NRAS-inducible HME-hTERT cells were cultured without tamoxifen, CDH1 downregulation increased the number of cells staining positive for PI, indicating induction of apoptosis. Tamoxifen-induction of CA-NRAS decreased the number of PI-positive cells only in the cells expressing control shRNA, while those cells expressing CDH1 shRNAs showed the same high population of PI staining cells in spite of CA-NRAS expression, suggesting that CDH1 is required for survival of HME-hTERT cells in soft agar independent of mutant-RAS (Figure 3D). Together, these results suggest that when non-malignant HME-hTERT cells are placed in an anchorage-independent growth condition, upregulation of CDH1 is required for the cells’ survival; the expression of mutant-RAS can only support anchorage-independent proliferation in the presence of sufficient expression of CDH1.

### 3.4. Expression of SNAI1/Snail, a Major EMT Regulator, Potentiates Malignant Transformation of HME-hTERT Cells by Mutant-RAS

**Figure 4:**
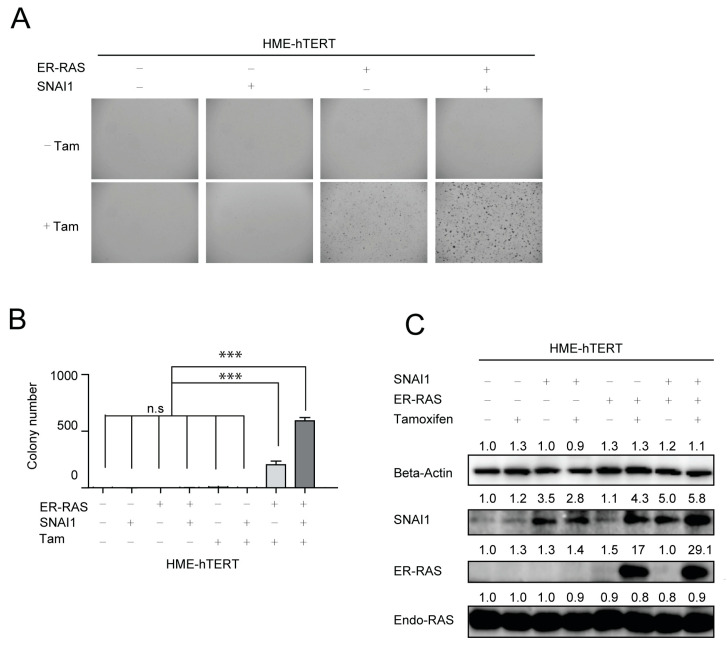
Mutant-RAS-driven cell proliferation in soft agar requires the upregulation of epithelial-mesenchymal transition (EMT). (**A**) Representative microscopic images of HME-hTERT cells grown in soft agar with or without the exogenous expression of CA-NRAS and/or SNAI1. (**B**) Quantitation of the data from panel A. Data are presented as mean ± SD of the number of colonies counted in three separate fields from each condition *** *p* ≤ 0.001, n.s: Not Significant, (Student’s *t*-test). The study presented has been repeated with similar results. (**C**) Immunoblot analysis of the indicated proteins in samples extracted from soft-agar cultures of HME-hTERT cells, which are cultured either in the presence or absence of exogenous expression of SNAI1 and/or CA-NRAS, as indicated.

Loss of CDH1/E-cadherin is often also observed during the induction of EMT, which can be achieved through the expression of major regulators of EMT such as SNAI1/Snail [36,37]. As direct knockdown of CDH1 in HME-hTERT cells resulted in increased apoptosis instead of increased colony-formation potential in soft agar, we proceeded to investigate the effect of promoting EMT, driven by SNAI1 overexpression, in the malignant transformation of HME-hTERT cells by mutant-RAS. To this end, we established stable SNAI1 expression and tamoxifen-inducible CA-NRAS (ER-CA-NRAS) expression in HME-hTERT cells. The resultant HME-hTERT and HME-hTERT + SNAI1 cells, with or without ER-CA-NRAS, were seeded in the soft agar in the presence or absence of tamoxifen (Figure 4A,B). Immunoblot analysis confirmed the elevated expression of SNAI1 and tamoxifen induction of CA-NRAS in HME-hTERT cells (Figure 4C). Expression of SNAI1 alone in HME-hTERT cells did not result in colony formation (Figure 4A,B). When CA-NRAS was induced, both HME-hTERT and HME-hTERT + SNAI1 cells formed colonies, but HME-hTERT + SNAI1 cells grew significantly more colonies (Figure 4A,B). Despite that SNAI1 is incapable of inducing colony formation on its own, it enhances CA-NRAS-induced colony formation in HME-hTERT cells, which is strong evidence that EMT is essential in setting the stage for mutant-RAS malignant transformation in this HMEC-based model.

### 3.5. Similar to SNAI1, SV40 Small T Antigen Induces EMT Changes in HME-hTERT Cells, Alleviates Dependency on CDH1 for Survival and Enhances Mutant-RAS-Induced Anchorage-Independent Colony Formation

**Figure 5:**
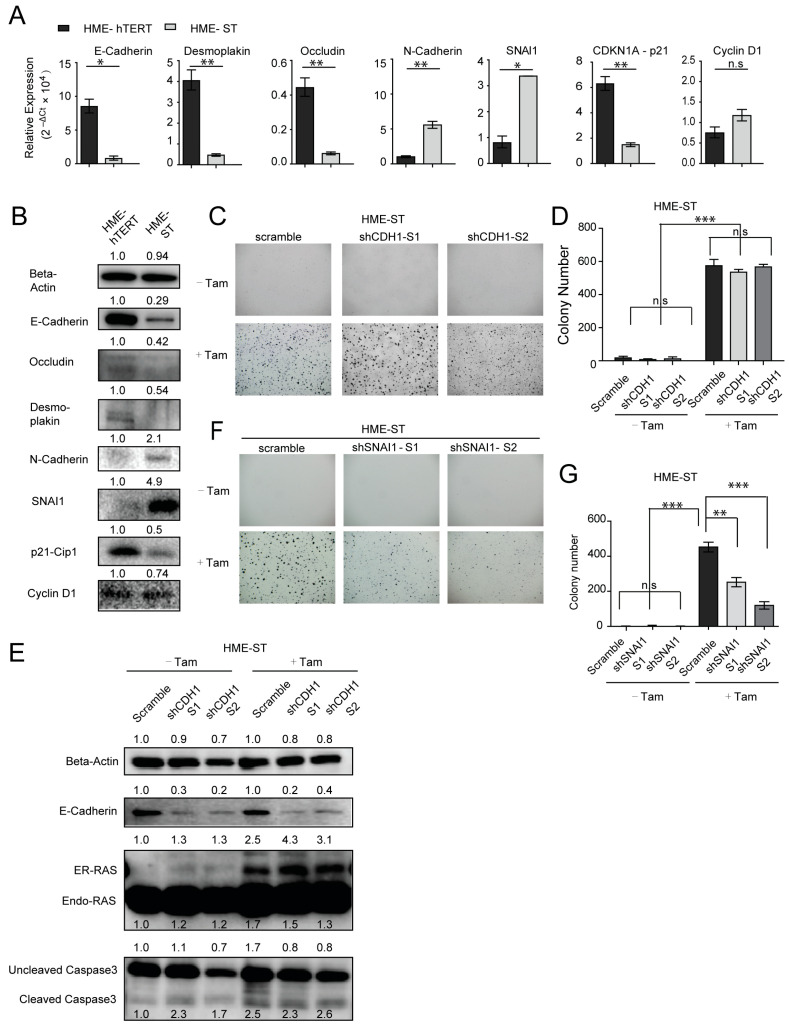
SV40 small T antigen functions to induce EMT in HME-hTERT cells and enables the cells to survive and grow in soft agar independent of CDH1. (**A**) RT-PCR analysis of the indicated genes was performed on RNA samples extracted from HME-hTERT and HME-ST cells grown in anchorage-independent soft-agar colony formation assays (CFAs). Statistical significance is reported as *p* values computed using mean −ΔCt values and normalized SD; ** *p* ≤ 0.01; * *p* ≤ 0.05, n.s: Not Significant. Data shown are the relative expressions ((2^−ΔCt^ × 10^4^) ± normalized SD) from duplicate determinations of a single experiment that is representative of three such experiments. (**B**) Corresponding immunoblot analysis on samples similarly prepared as in panel A to evaluate the levels of the indicated proteins; samples were extracted from cells grown in soft agar. (**C**) Representative microscopic images of soft-agar CFAs of HME-ST cells expressing either a control shRNA (scramble) or two different shRNAs targeting CDH1, with or without tamoxifen-induced expression of CA-NRAS. (**D**) Quantitation of the data from the experiment shown in panels C; data are presented as mean ± SD numbers of colonies counted in three separate fields. (**E**) Immunoblot analysis of protein samples extracted from cells in soft agar corresponding to the conditions of panels C and D. (**F**) Representative microscopic images of soft agar colonies of HME-ST cells with or without expressing CA-NRAS and with the expression of either a control shRNA or two different shRNAs targeting SNAI1. (**G**) Quantitation of the data presented in panel F; data are presented as mean ± SD. ** *p* ≤ 0.01, *** *p* ≤ 0.001 (Student’s *t*-test).

As presented in Figure 1B, ST expression alone in HME-hTERT cells does not lead to anchorage-independent colony formation; however, ST expression enhances the effect of mutant-RAS on anchorage-independent colony formation, in a way similar to the action of SNAI1. Hence, we investigated whether ST expression would lead to EMT changes in HME-hTERT cells when cultured in soft agar. Using the RNA and protein samples extracted from cells cultured in soft agar, both q-RT-PCR and Western blot studies showed that ST expression resulted in remarkable downregulation of epithelial markers CDH1, OCLN and DSP and significant upregulation of mesenchymal markers CDH2 and SNAI1 (Figure 5A,B). These findings strongly suggest that ST expression induces EMT-related changes. Comparison of RNA and protein levels of CDKN1A (p21^Cip1^) between HME-hTERT and HME-ST cells also showed that CDKN1A is downregulated when ST is expressed (Figure 5A,B), supporting the notion that HME-ST cells are primed for anchorage-independent proliferation although they are still unable to do so in the absence of mutant-RAS (Figure 1A,B). Further supporting this, RNA and protein expression levels of CCND1 (Cyclin D1) were not significantly affected by ST expression (Figure 5A,B), suggesting that the expression of ST alone merely primes HME cells but does not make them actively proliferative in anchorage-independent soft-agar CFAs.

Since ST expression leads to EMT-related changes and enhanced colony formation upon mutant-RAS expression, we hypothesize that, in contrast to HME-hTERT cells, acquiring ST renders the cells to be in a transition state that is no longer dependent on CDH1 for survival when cultured in soft agar. To test this hypothesis, we established stable knockdown of CDH1 in HME-ST cells that can express RAS upon tamoxifen induction. We found that, in these HME-ST cells, CDH1 knockdown had no significant effect on the colony formation stimulated by CA-RAS expression (Figure 5C,D), in stark contrast to the effect of knocking down CDH1 on HME-hTERT cells (Figure 3A,B). Western blot analysis showed that CDH1 knockdown in HME-ST cells did not impact the levels of cleaved caspase 3, suggesting that there is no change in the apoptosis status in either of the cell lines (Figure 5E), in contrast to HME-hTERT cells (Figure 3C,D), suggesting that ST expression indeed abolishes HME cells’ dependency on CDH1 for survival in anchorage-independent culture.

We seek to provide further evidence that the mechanism behind ST priming HME cells for mutant-RAS transformation is via promoting EMT. To this end, we knocked down the established EMT regulator SNAI1 in HME-ST cells to investigate the role of EMT in mutant-RAS-driven transformation in HME-ST cells. We found that HME-ST cells showed a significant reduction of mutant-RAS-induced colony formation when SNAI1 was downregulated (Figure 5F,G). Together, these data firmly establish that EMT plays a critical role in anchorage-independent survival and colony formation of HME cells, predisposing the cells for further transformation and eliminating cell dependency on adhesion for survival. Specific to the HME model in the study, the evidence supports the critical role of SV40 small T antigen in effectively priming HME-hTERT cells for anchorage-independent growth by inducing EMT.

## 4. Discussion

A hallmark for transformed cells is their ability to proliferate under anchorage-independent conditions. Therefore, the cellular signaling events that govern transformation should, and at times necessarily, be analyzed in cells grown under such conditions. Anchorage-independent colony formation assays have been widely used as an in vitro surrogate assay to assess the tumorigenic potential of cell lines, especially in pre-clinical drug development due to its low cost and fast turnaround time. However, the major limitation of the soft-agar CFA is that it is generally an end-point assay that does not readily allow for mechanistic analyses of the molecular processes involved. Although approaches to pick and analyze individual colonies have been reported, they are limited by selection bias and the small amount of available material, and these protocols cannot be used in situations when no discernible colony forms, as in cases of drug treatment or genetic manipulation that inhibits anchorage-independent growth [4,5].

To tackle the problem of mechanistic analyses of cancer cells embedded in soft agar, we established a cetyltrimethylammonium chloride (CTAC)-based method to extract high-quality RNA and DNA from cells embedded in soft agar (see Section 2 and Supplementary Materials Figure S2). Validation experiments showed that the DNA and RNA obtained using this protocol are of sufficiently high quality to be used in demanding next-generation sequencing protocols, as well as reproducible q-RT-PCR analysis (see Section 2 and Figure S2). This CTAC-based extraction method does not require any specialized equipment beyond that found in most biology laboratories, and the reagents required can be easily procured at a reasonable cost. Most importantly, in our experience, the high quality of the RNA and DNA obtained using this protocol is highly reproducible, providing the confidence to use the material obtained for mechanistic analyses.

Beyond DNA and RNA analysis, the analysis of functionally relevant cellular events would not be complete without quantifying proteins, particularly the post-translational modification of proteins such as phosphorylation. However, this too has been a challenge in cells cultured in soft-agar matrices. Hence, we also developed a method that enables the extraction of proteins from cells embedded in soft agar that retain their modification statuses. Using well-characterized mTOR inhibitor Rapamycin, we demonstrated that this protocol can be reliably used for quantifying proteins and phosphoproteins that inform upon specific regulatory pathways in cells grown in soft agar (Figure S2).

Factors involved in epithelial-mesenchymal transition (EMT) and their roles have been subjected to extensive evaluation. Growth factors and transcription factors promoting EMT such as TGF-β, SNAI1/2, ZEB1/2 and TWIST1 support transformation in cooperation with other oncogenic factors [21,22]. EMT has been shown to play a crucial role in tumorigenesis driven by mutant-RAS [26]. Curiously, despite a long history of using HME cells’ stepwise transformation as a model for studying cancer development, the contributions of the individual oncogenic factors, such as that by SV40 small T (ST) antigen, in relation to EMT, have not been explicitly evaluated. As most gene expression analysis so far has been performed using the materials obtained from cells cultured under the adherent condition, it is possible that the manifestation of EMT changes caused by the expression of these oncogenic factors could not be evaluated in this setting. The newly-developed macromolecule extraction methods have now made it possible to analyze these signaling alterations in cells embedded in soft agar. As a demonstration of the utility of our methods, we made discoveries using HME cell lines that are only possible by analyzing cells growing in the anchorage-independent soft-agar CFAs. In addition to HME cells, we found the method is a useful tool to study cancer cell signaling changes in response to the therapeutics (Figure S2F—Phosphoprotein analysis in MiaPaCa2 pancreatic cells).

The loss of E-cadherin (CDH1) expression is widely accepted as one of the most important hallmarks of EMT, and it has also been suggested that loss of E-cadherin signifies increased survival for cancer cells [38]. On the other hand, it has also been reported that E-cadherin expression is essential for aggregation-dependent cell survival [39,40,41]. We found that, for immortal HME-hTERT cells that are unable to form colonies in soft agar, upregulation of CDH1 is essential for cell survival and subsequent oncogenic RAS-driven proliferation in anchorage-independent conditions (Figure 3). This result is consistent with the notion that anchorage/adhesion driven by CDH1, both to attachment surfaces and to each other, is important for the survival and proliferation of cells that have not achieved full malignant potential. This finding, which can only be discovered by the analysis of the cells grown in anchorage-independent conditions, strengthens the role of CDH1 expression for cells to evade anoikis in anchorage-independent conditions.

Acquiring mesenchymal characteristics is part of the transformation process to do away with the need for anchorage for survival and gain resistance to anoikis. SV40 small T antigen is a classic oncogene used in in vitro experiments that study the processes of oncogenesis. In this study, we gathered evidence to show that the expression of ST in HME-hTERT cells also results in the upregulation of EMT transcription regulator SNAI1 and other EMT-related changes in soft-agar CFAs, such as downregulation of E-cadherin, occludin and desmoplakin and the upregulation of N-cadherin, and an increased propensity to form anchorage-independent colonies induced by mutant-RAS. The same analysis on cells grown in adherent culture do not show any clear difference in SNAI1 expression before and after the introduction of ST, nor does the expression of mutant-RAS increase the level of SNAI1 (Figure S3). The comparison of the protein markers between adherent and soft-agar cultures demonstrates that some of the most important EMT marker changes only manifest when the cells are growing in anchorage-independent conditions, which underscores the necessity of evaluating signaling changes under experimental conditions that are relevant to the questions addressed.

## 5. Conclusions

Using the novel macromolecule extraction methods that we developed, we were able to study the relevant cell signaling events contributing to the priming of HME cells for anchorage-independent growth in soft agar, which allowed us to confirm two important conclusions that were not possible from studies of adherent cells. First, we found that while HME-hTERT cells depend on adhesion molecules for survival under anchorage-independent conditions, acquisition of further oncogenic stimuli enables anchorage-independent growth and colony formation by up-regulating the EMT program. Further, we established the fundamental role of EMT in ST-driven enhancement of oncogenic potential in the HMEC transformation model.

The soft-agar CFA remains the most relevant assay for the in vitro assessment of tumorigenicity. Through the questions addressed in this study, we also demonstrated the validity and utility of the new macromolecule extraction methods described for the analysis of molecular signatures of cells embedded in soft agar, and they should be of significant benefit to the scientific community, especially in drug development research.

## Data Availability

No new data sets were created or analyzed in this study. Data sharing is not applicable to this article.

## Supplementary Materials

The following are available online at https://www.mdpi.com/2072-6694/13/4/807/s1, Figure S1: RT-PCR analysis performed on HME-hTERT and HME-ST cells expressing exogenous mutant isoforms of RAS (+) or empty vector control (−) using primers against exogenous KRAS-4B, NRAS and HRAS, Figure S2: Detection of changes in DNA, RNA and protein levels using the samples extracted from cells cultured in soft agar using the new extraction protocol, Figure S3: Western blot analysis performed using protein samples extracted from 2D adherent cultures of HME-hTERT and HME-ST, with or without introducing exogenous SNAI1, in the presence or absence of tamoxifen induced CA-NRAS expression, Figure S4: Original western blots, Table S1: Sequences of primers used for quantitative real-time polymerase chain reaction.
