# Supplementary material for: Evaluating the Epithelial-Mesenchymal Program in Human Breast Epithelial Cells Cultured in Soft Agar Using a Novel Macromolecule Extraction Protocol
Source: Cancers (Basel). 2021 Feb 15;13(4):807. doi: 10.3390/cancers13040807 (PMC7919038; doi:10.3390/cancers13040807)
Supplement: Supplementary file 1 [file cancers-13-00807-s001.zip › cancers-1064233-supplementary.pdf]

cancers

MDPI

Supplementary Materials

Evaluating the Epithelial-Mesenchymal Program in Human Breast Epithelial Cells Cultured in Soft Agar Using a Novel Macromolecule Extraction Protocol

Hiu Yeung Lau , Jingyi Tang , Patrick J. Casey and Mei Wang

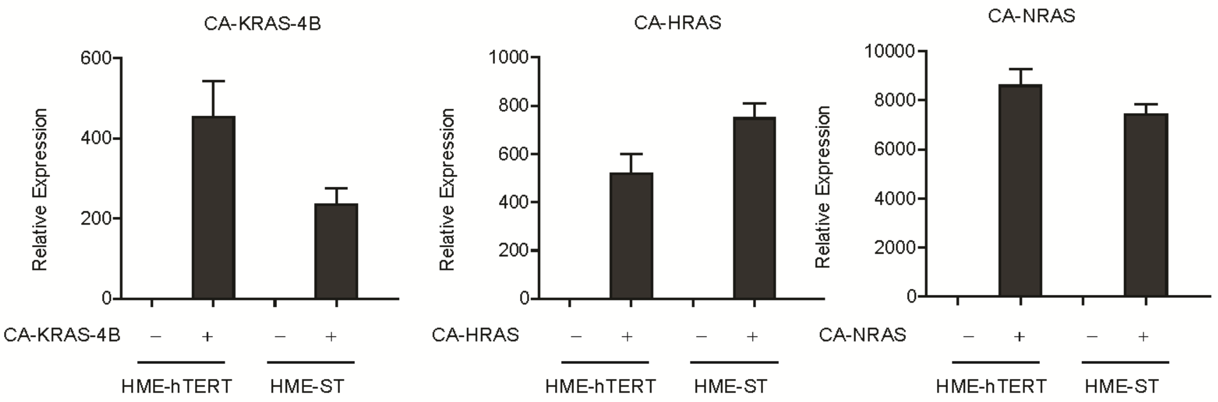

Figure S1. RT-PCR analysis performed on HME-hTERT and HME-ST cells expressing exogenous mutant isoforms of RAS (+) or empty vector control (–) using primers against exogenous KRAS-4B, NRAS and HRAS.

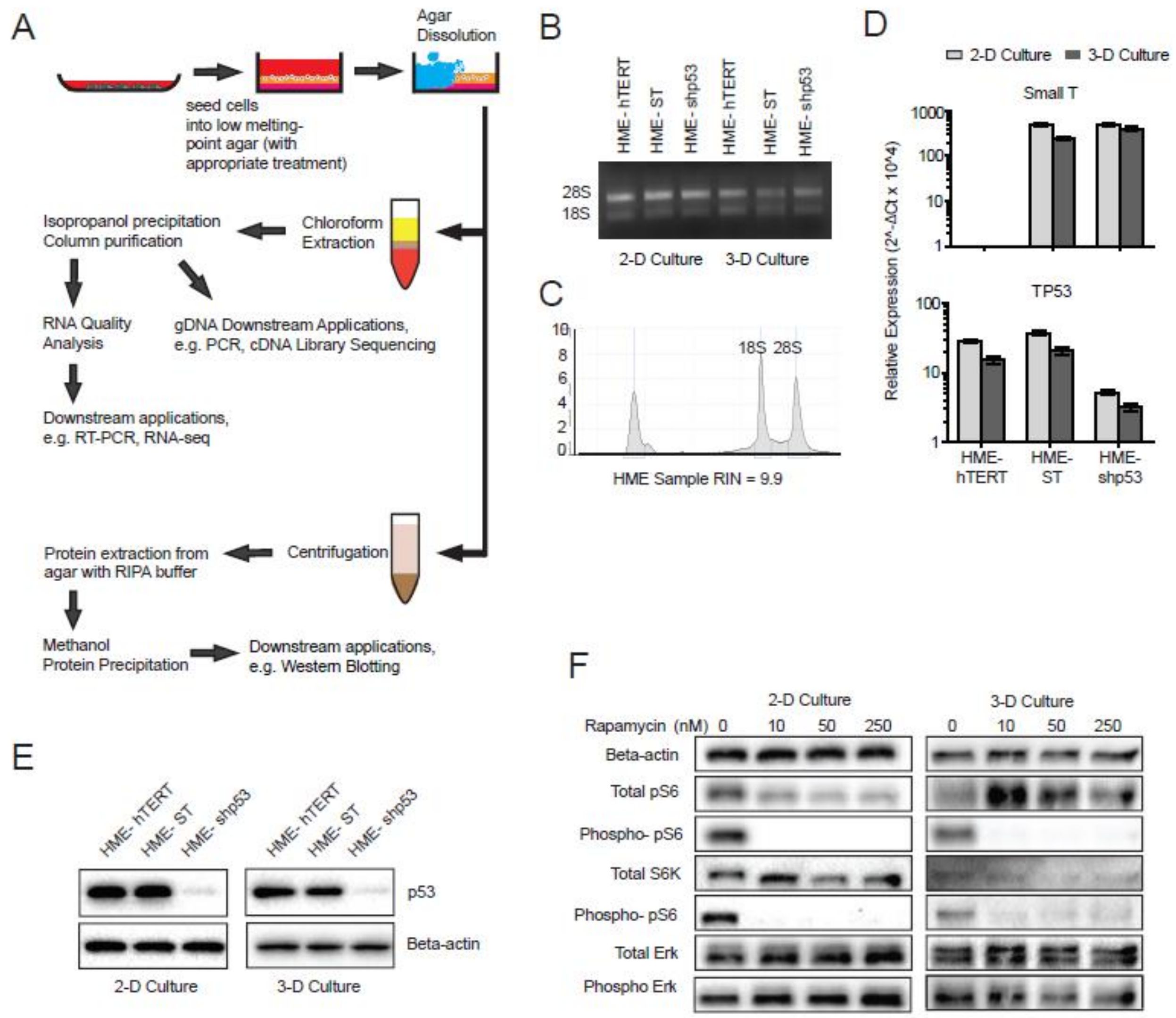

Figure S2. Detection of changes in DNA, RNA and protein levels using the samples extracted from cells cultured in soft agar using the new extraction protocol. (**A**) Illustration of the workflow for both the CTAC-based RNA and DNA extraction protocol and the chloroform-methanol based protein extraction protocol from cells grown in soft-agar. (**B**) Agarose-gel electrophoresis of RNAs extracted from the indicated cell lines demonstrating that the CTAC-based protocol is able to extract intact RNAs from soft-agar (3-D) (right side) with comparable quality to the RNA samples from cells in adherent culture (2-D) (left side). (**C**) RNA integrity analysis Agilent Technologies 2100 Bioanalyzer and the results reported as RNA Integrity Number (RIN). (**D**) RT-PCR analysis using RNA samples extracted from the indicated HME cells cultured under conventional adherent (2-D, grey bar) as well as in soft agar (3-D, dark grey bar) conditions. Both sets of RNA samples reliably reflect the expected expression of small T antigen and downregulation of p53 in HME-hTERT, HME-ST and HME-shp53 cells. (**E**) Immunoblot analysis for p53 expression in HME-hTERT, HME-ST and HME-shp53 cells using Beta-actin as loading control. (**F**) Immunoblot analysis of the indicated proteins and phosphoproteins extracted from MiaPaCa-2 cells grown under adherent (2-D) and soft agar (3-D) conditions after subjected to Rapamycin treatment at escalating concentrations; the protein samples from soft agar are extracted using the newly-developed protocol.

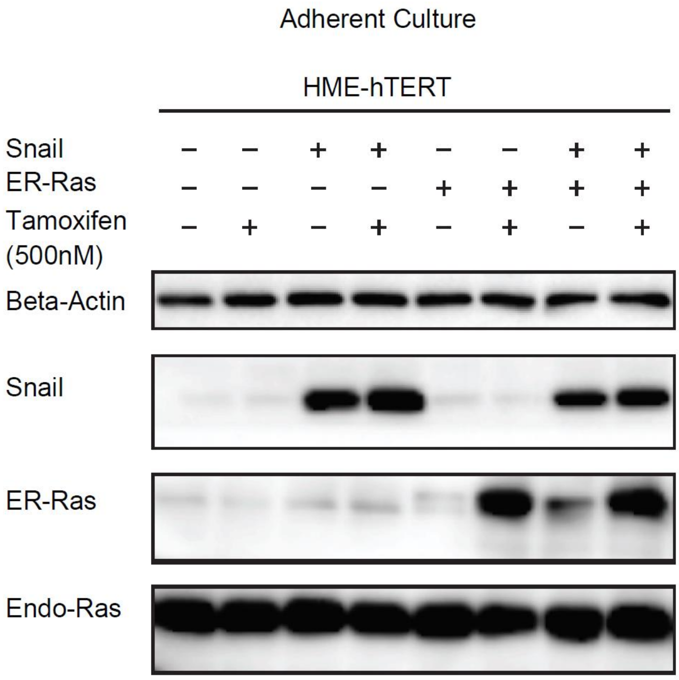

Figure S3. Western blot analysis performed using protein samples extracted from 2D adherent cultures of HME-hTERT and HME-ST, with or without introducing exogenous SNAI1, in the presence or absence of tamoxifen induced CA-NRAS expression.

Table S1. Sequences of the primers used in q-RT-PCR.

RT-PCR primer	Forword	Reverse
SNAL1	CCAGTGCCTCGACCACTATG	CTGCTGGAAGGTAAACTCTGG
SNAL2(SLUG)	CACTGCGATGCCCAGTCTA	TTCTCCCCCGTGTGAGTTCTAA
ZEB1	AGTGGTCATGATGAAAATGGAAC	AGGTGTAACTGCACAGGGAGC
ZEB2	CAACCATGAGTCCTCCCCAC	GTCTTCCTTCATTTCTTCTGGACC
Occludin	AAAGTCCACCTCCTTACAGGC	GGCTGAGAGAGCATTGGTCG
CDH2	AGGCTTCTGGTGAAATCGCA	TGCAGTTGCTAAACTTCACATTG
CDH1	GTCCTGGGCAGAGTGAATTT	GACCAAGAAATGGATCTGTGG
CDKN1A	CTGTGATGCGCTAATGGCG	AAGTCGAAGTTCCATCGCTCA
CyclinD1	AACTACCTGGACCGCTTCCT	CCACTTGAGCTTGTTCACCA
Fibronectin	CTGGCCAGTCCTACAACCAG	CGGGAATCTTCTCTGTCAGCC
Vimentin	CGAGGAGAGCAGGATTTCTC	GGTATCAACCAGAGGGAGTGA
TP53	ACCTATGGAAACTACTTCCTGAAA	CTGGCATTCTGGGAGCTTCA
Desmoplakin	GGCACCAGCAGGATGTACTA	GATGAGCTCTGCTCGCATCA
**shRNA sequence**	**Targeting sequence**	
pLL3.7 shSNAi1_1	GCCATGGAATTCCCTCCTG	
pLL3.7 shSNAi1_2	GCCTAACTACAGCGAGCTG	
pLL3.7 shCDH1_1	GTGATGCAGTTAGTATAGC	
pLL3.7 shCDH1_2	GAACAGCACGTACACAGCC	
