# Supplementary material for: Evaluating the Epithelial-Mesenchymal Program in Human Breast Epithelial Cells Cultured in Soft Agar Using a Novel Macromolecule Extraction Protocol
Source: Cancers (Basel). 2021 Feb 15;13(4):807. doi: 10.3390/cancers13040807 (PMC7919038; doi:10.3390/cancers13040807)
Supplement: Supplementary file 1 [file cancers-13-00807-s001.zip › Figure S4 original western blots/Fig 5B - raw data.pptx]

## Slide 1
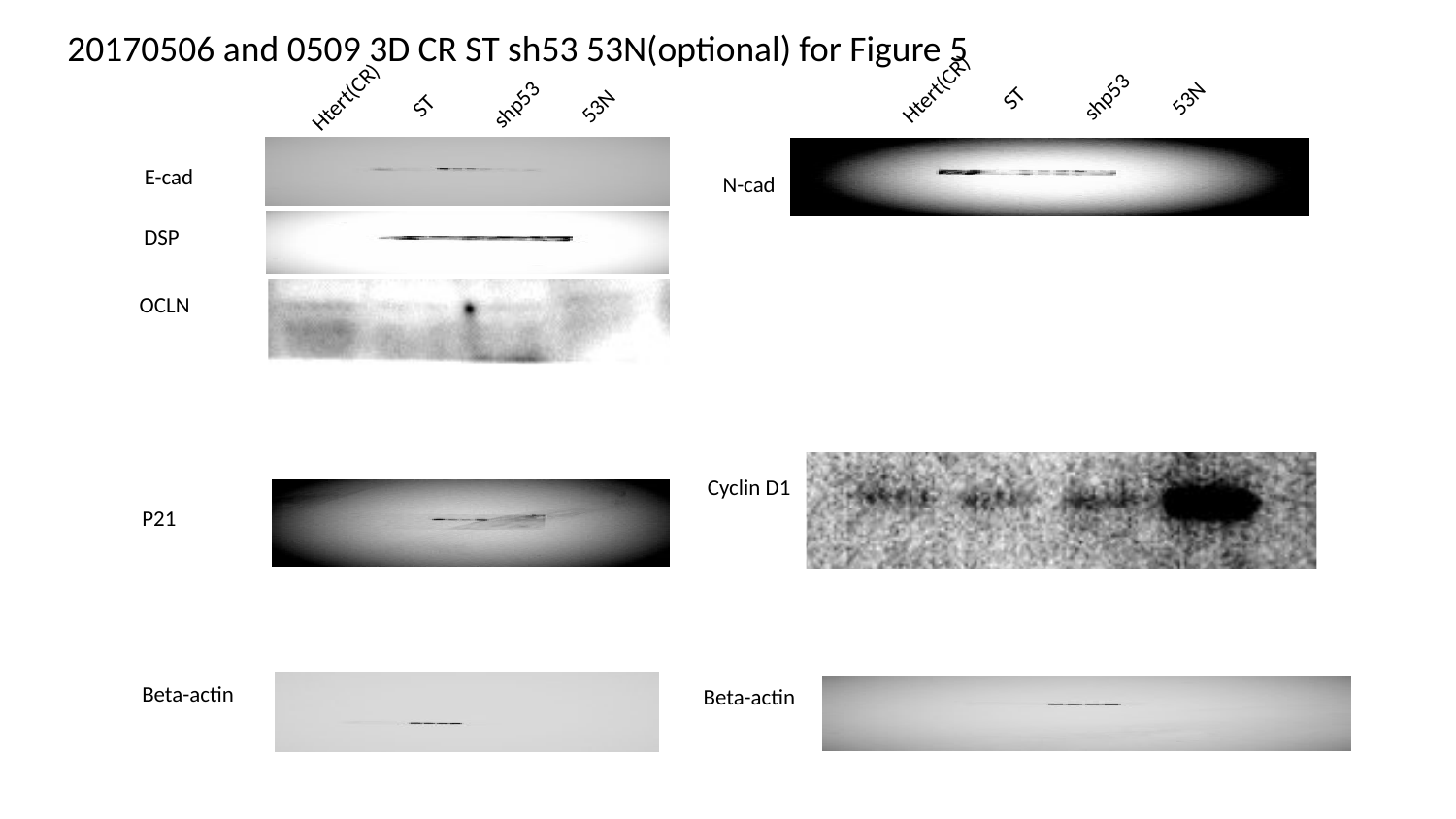

20170506 and 0509 3D CR ST sh53 53N(optional) for Figure 5
Htert(CR)
shp53
Htert(CR)
ST
53N
shp53
ST
53N
E-cad
N-cad
DSP
OCLN
Cyclin D1
P21
Beta-actin
Beta-actin
