# Supplementary material for: Evaluating the Epithelial-Mesenchymal Program in Human Breast Epithelial Cells Cultured in Soft Agar Using a Novel Macromolecule Extraction Protocol
Source: Cancers (Basel). 2021 Feb 15;13(4):807. doi: 10.3390/cancers13040807 (PMC7919038; doi:10.3390/cancers13040807)
Supplement: Supplementary file 1 [file cancers-13-00807-s001.zip › Figure S4 original western blots/Sub Fig 2F - raw gel.pptx]

## Slide 1
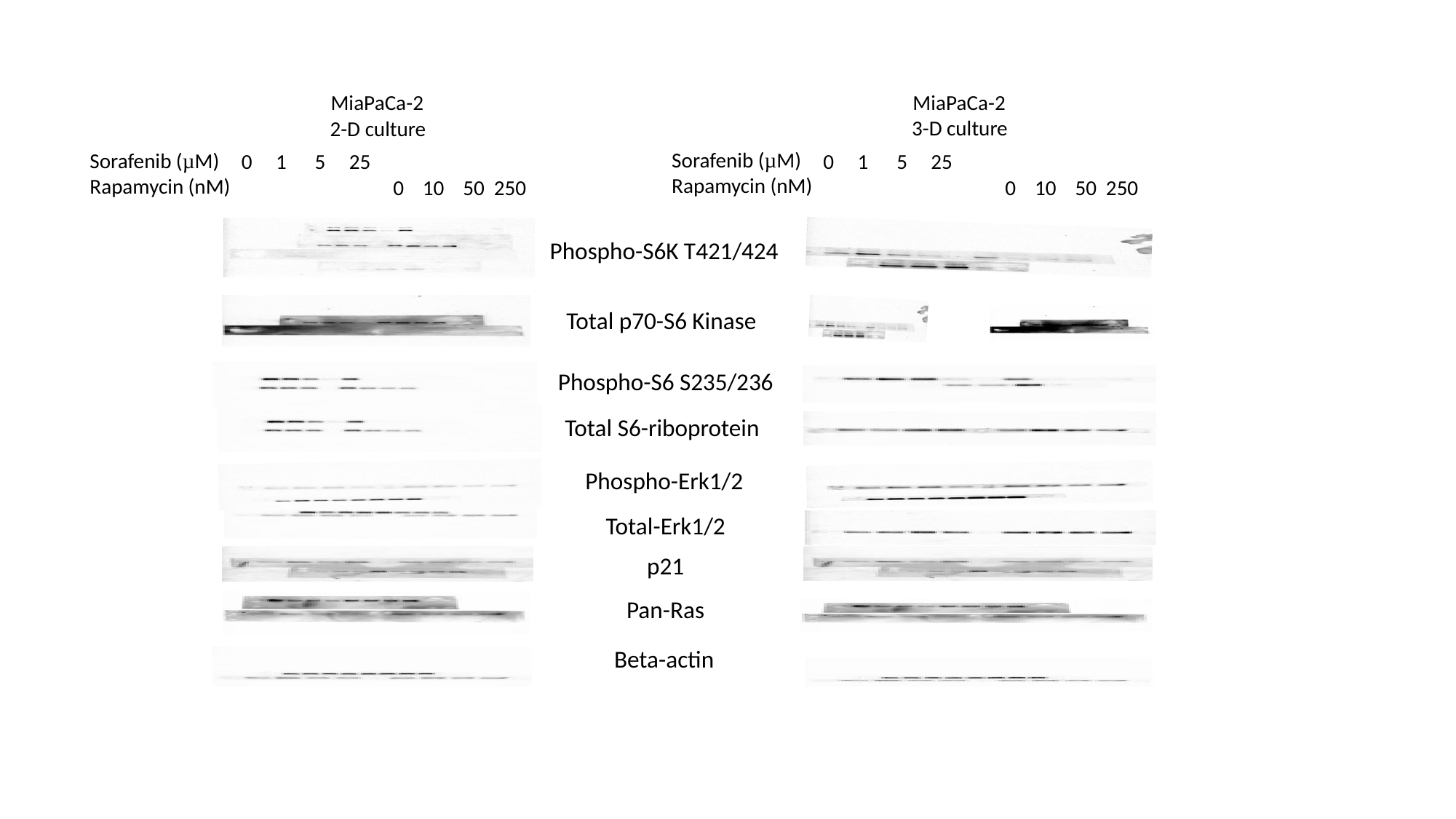

MiaPaCa-2
MiaPaCa-2
3-D culture
2-D culture
Sorafenib (µM)
Rapamycin (nM)
Sorafenib (µM)
Rapamycin (nM)
 0 1 5 25
 0 1 5 25
 0 10 50 250
 0 10 50 250
Phospho-S6K T421/424
Total p70-S6 Kinase
Phospho-S6 S235/236
Total S6-riboprotein
Phospho-Erk1/2
Total-Erk1/2
p21
Pan-Ras
Beta-actin
