# Supplementary figures and images for: Evaluating the Epithelial-Mesenchymal Program in Human Breast Epithelial Cells Cultured in Soft Agar Using a Novel Macromolecule Extraction Protocol
Source: Cancers (Basel). 2021 Feb 15;13(4):807. doi: 10.3390/cancers13040807 (PMC7919038; doi:10.3390/cancers13040807)

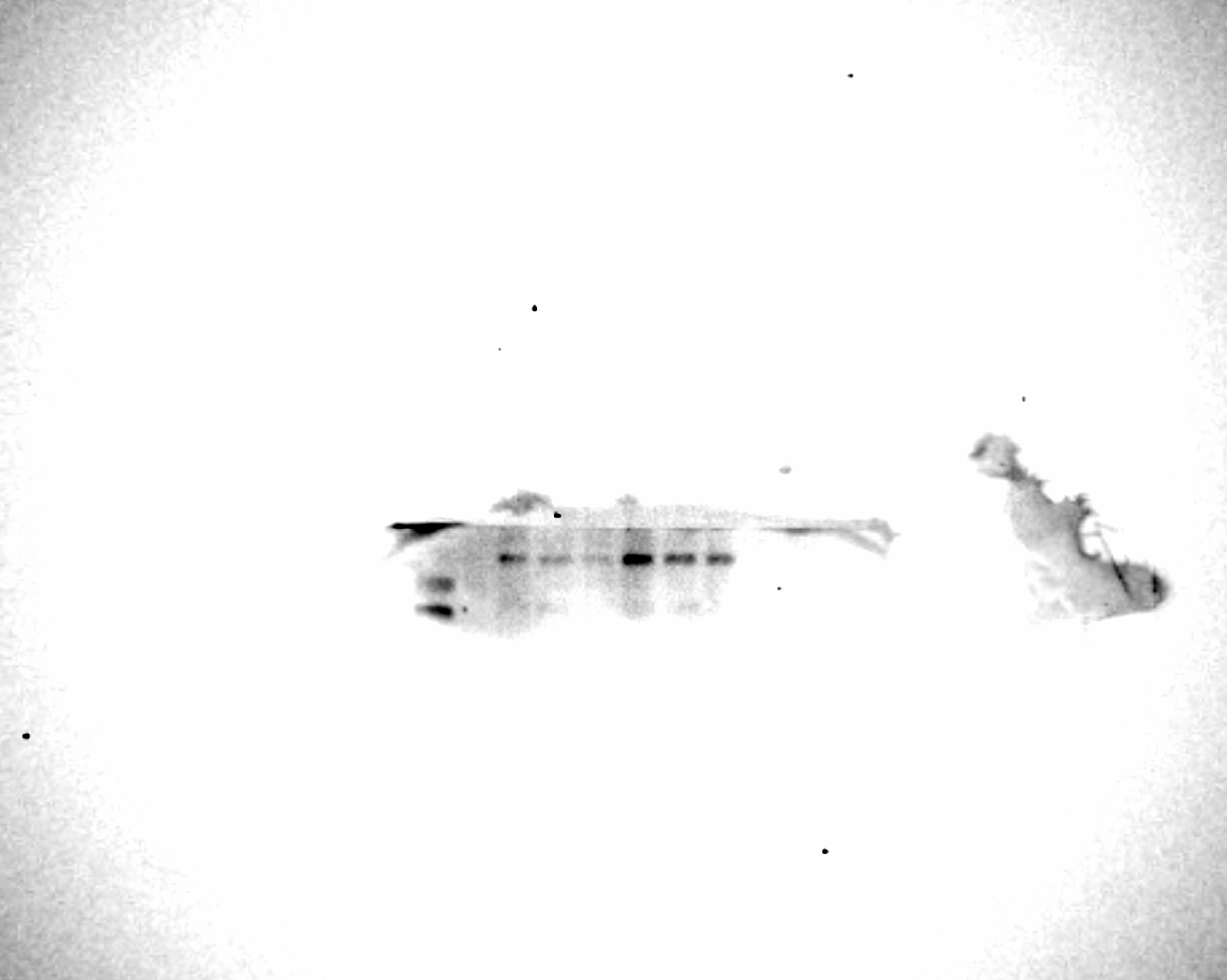

Supplement: Supplementary file 1 [file cancers-13-00807-s001.zip › Figure S4 original western blots/3C/3dstsnail_29.tif]

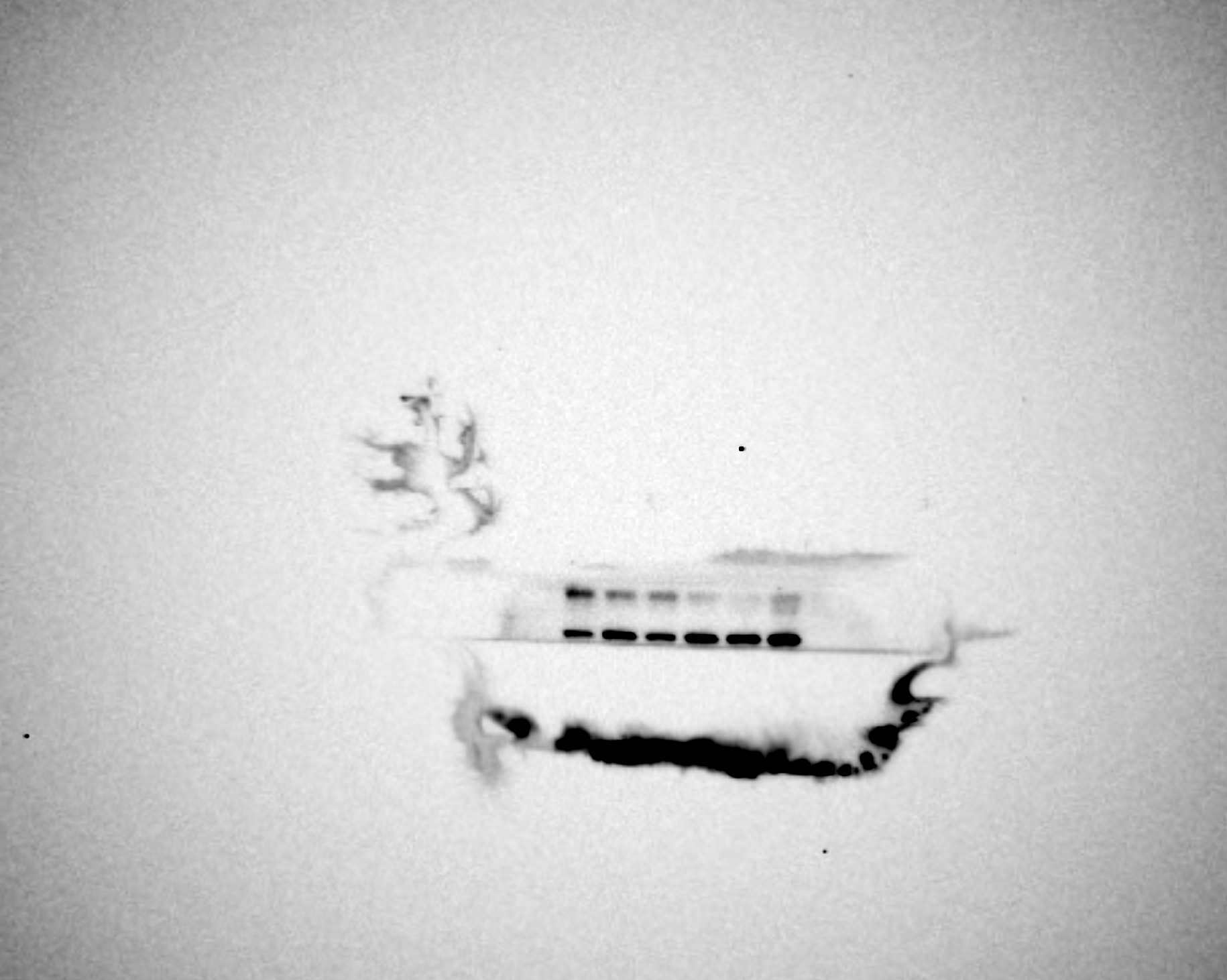

Supplement: Supplementary file 1 [file cancers-13-00807-s001.zip › Figure S4 original western blots/3C/actib3dst_07.tif]

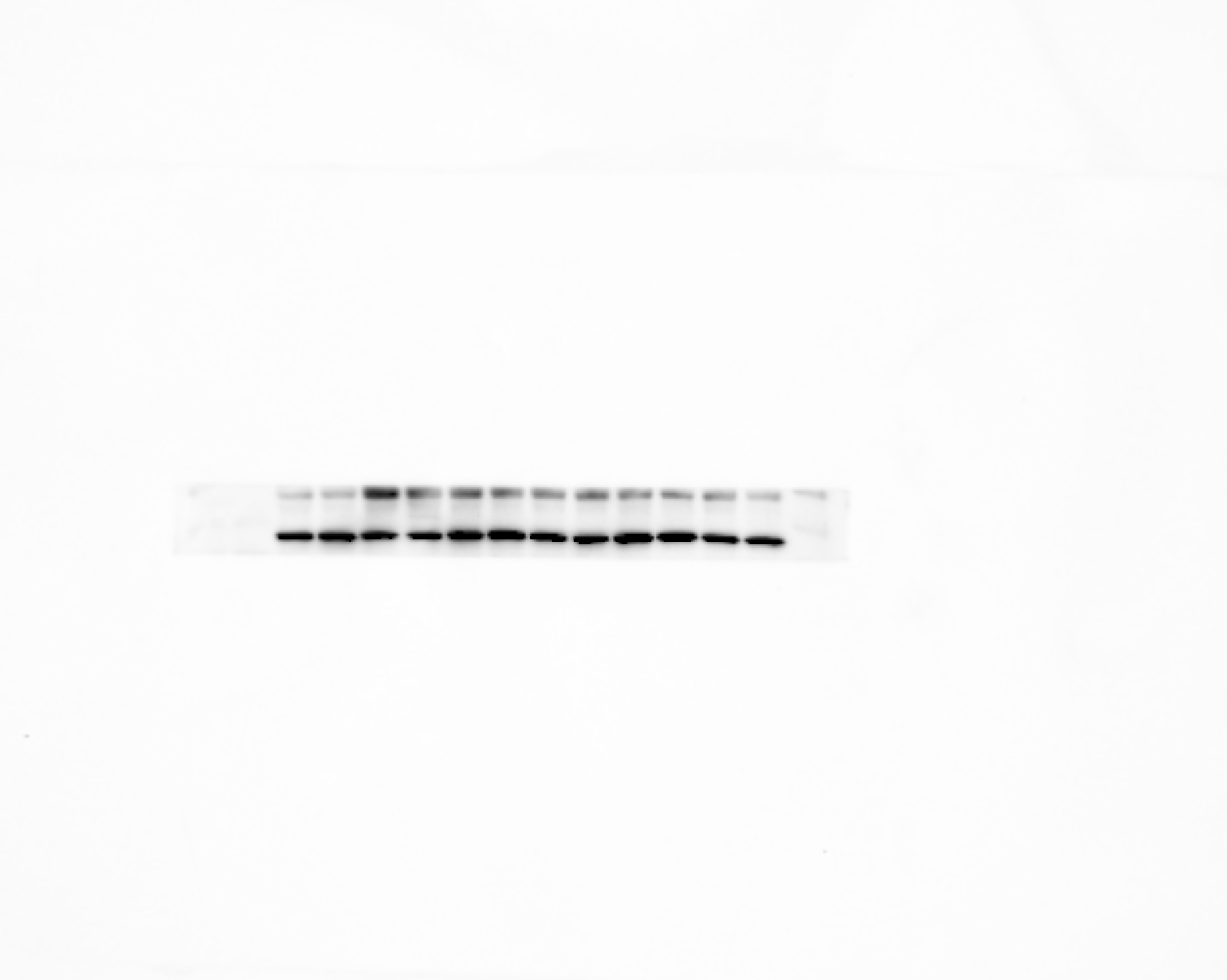

Supplement: Supplementary file 1 [file cancers-13-00807-s001.zip › Figure S4 original western blots/3C/actin1018_12.tif]

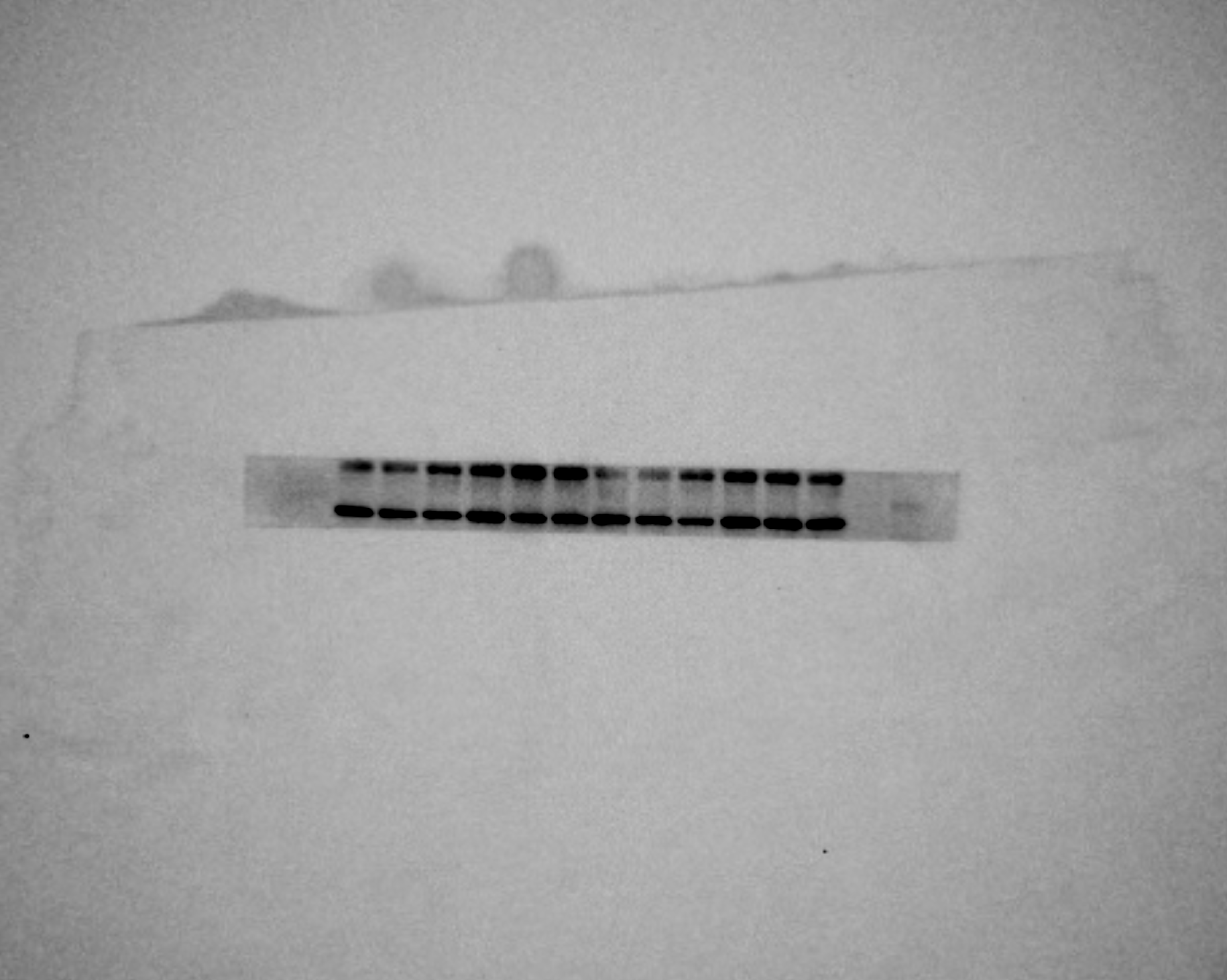

Supplement: Supplementary file 1 [file cancers-13-00807-s001.zip › Figure S4 original western blots/3C/acting1_34.tif]

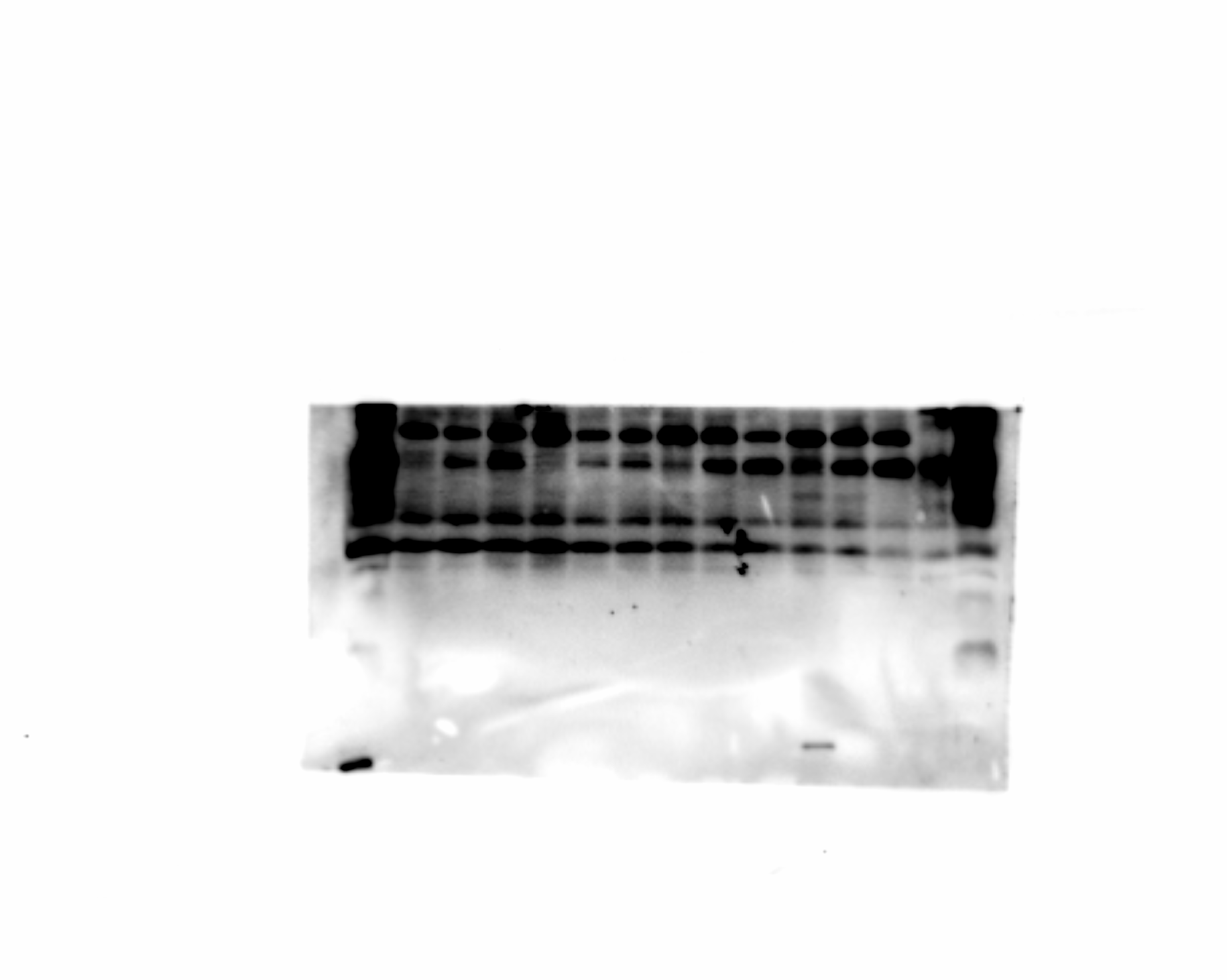

Supplement: Supplementary file 1 [file cancers-13-00807-s001.zip › Figure S4 original western blots/3C/cas3stcr3d_51.tif]

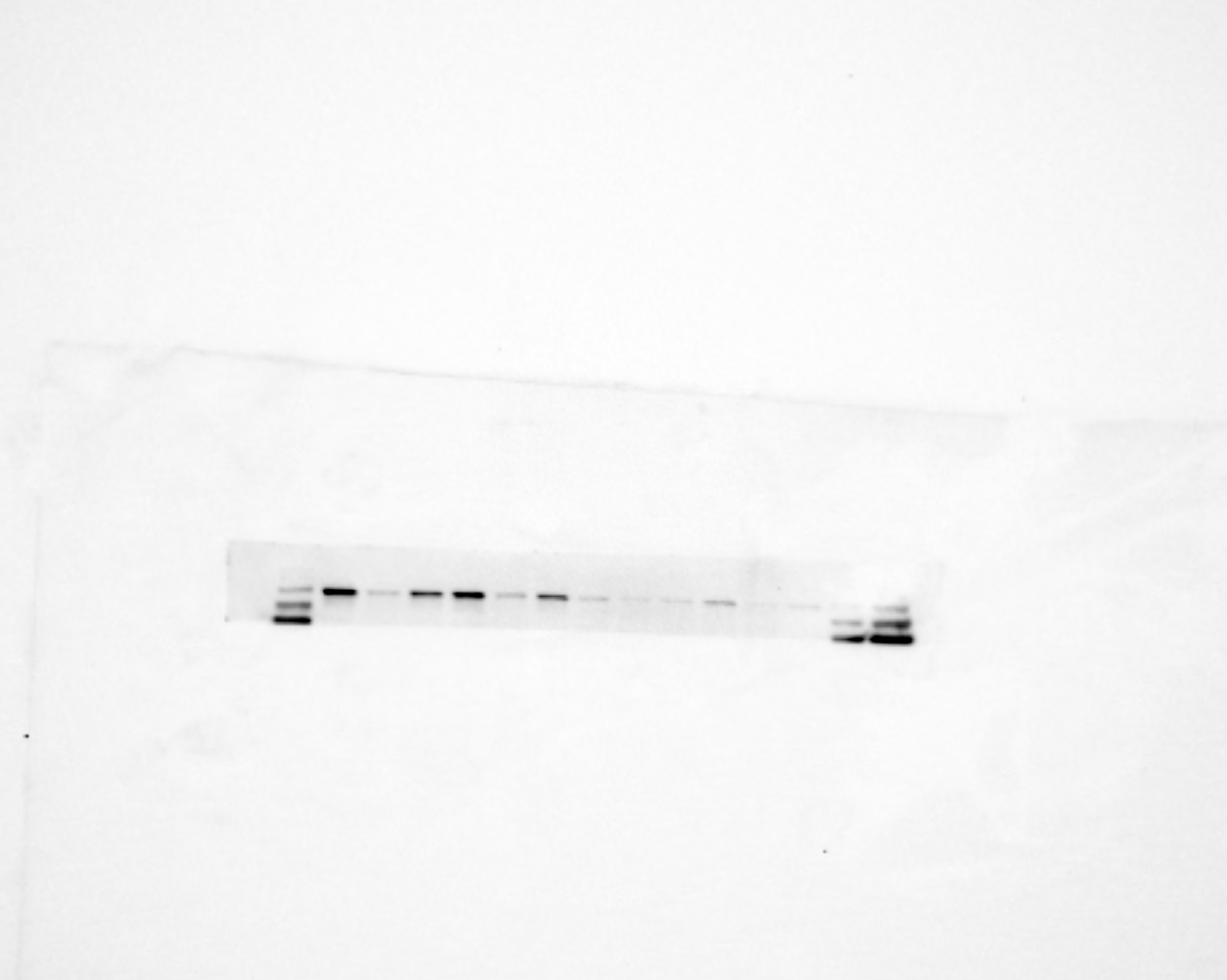

Supplement: Supplementary file 1 [file cancers-13-00807-s001.zip › Figure S4 original western blots/3C/ecad_61.tif]

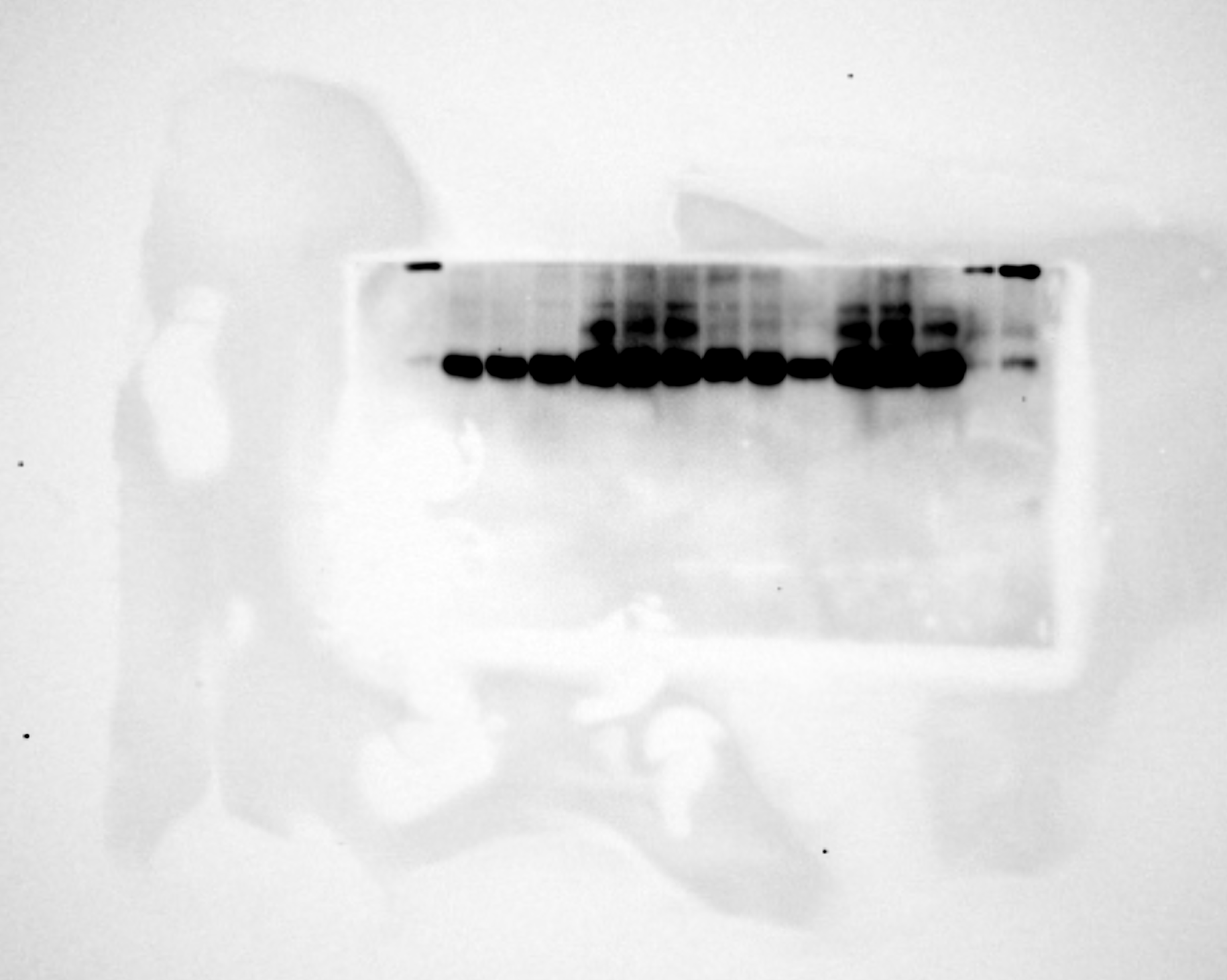

Supplement: Supplementary file 1 [file cancers-13-00807-s001.zip › Figure S4 original western blots/3C/g1ras_083.tif]

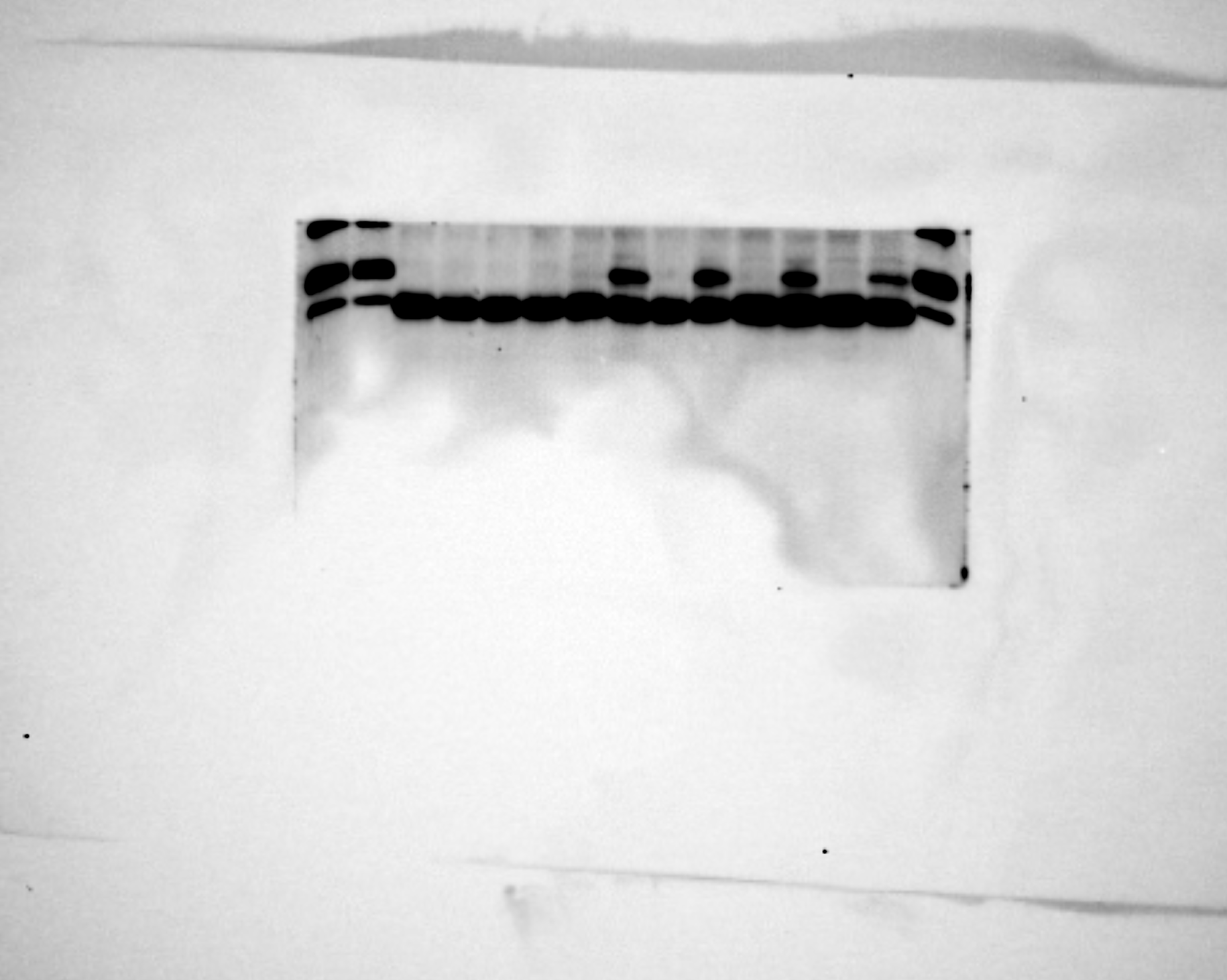

Supplement: Supplementary file 1 [file cancers-13-00807-s001.zip › Figure S4 original western blots/3C/ras-2_33.tif]

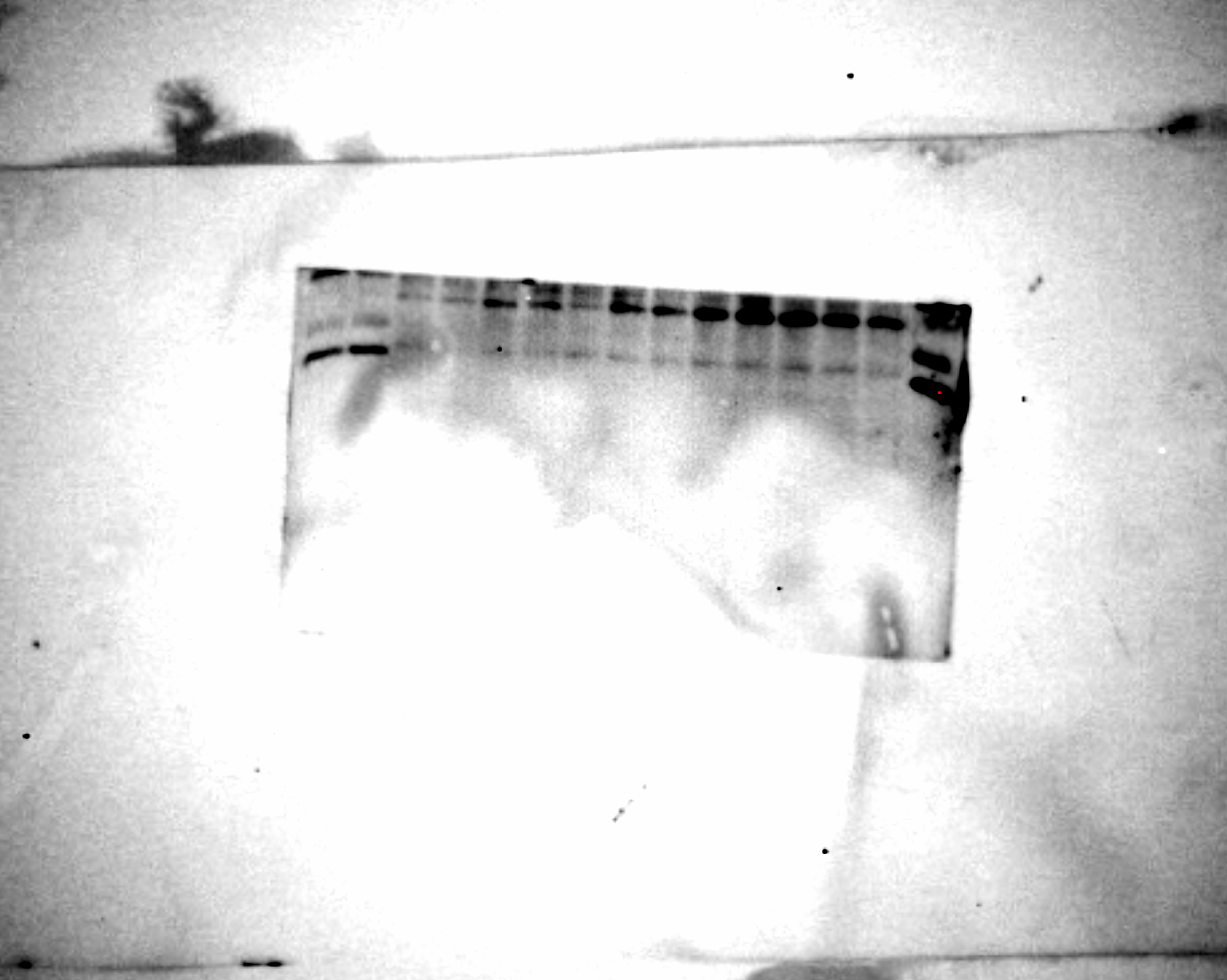

Supplement: Supplementary file 1 [file cancers-13-00807-s001.zip › Figure S4 original western blots/3C/snail1018_141.tif]

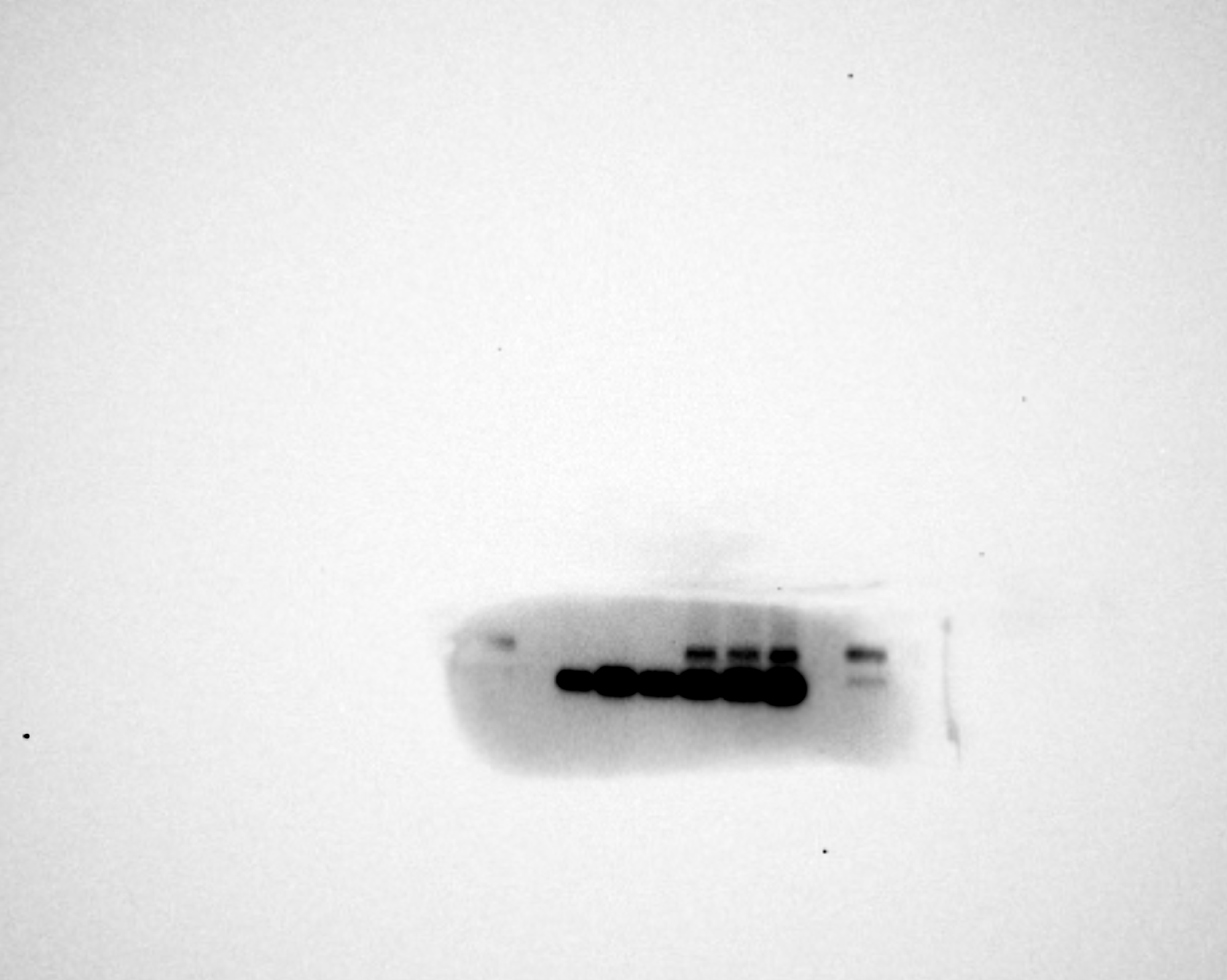

Supplement: Supplementary file 1 [file cancers-13-00807-s001.zip › Figure S4 original western blots/3C/stras3d_51.tif]

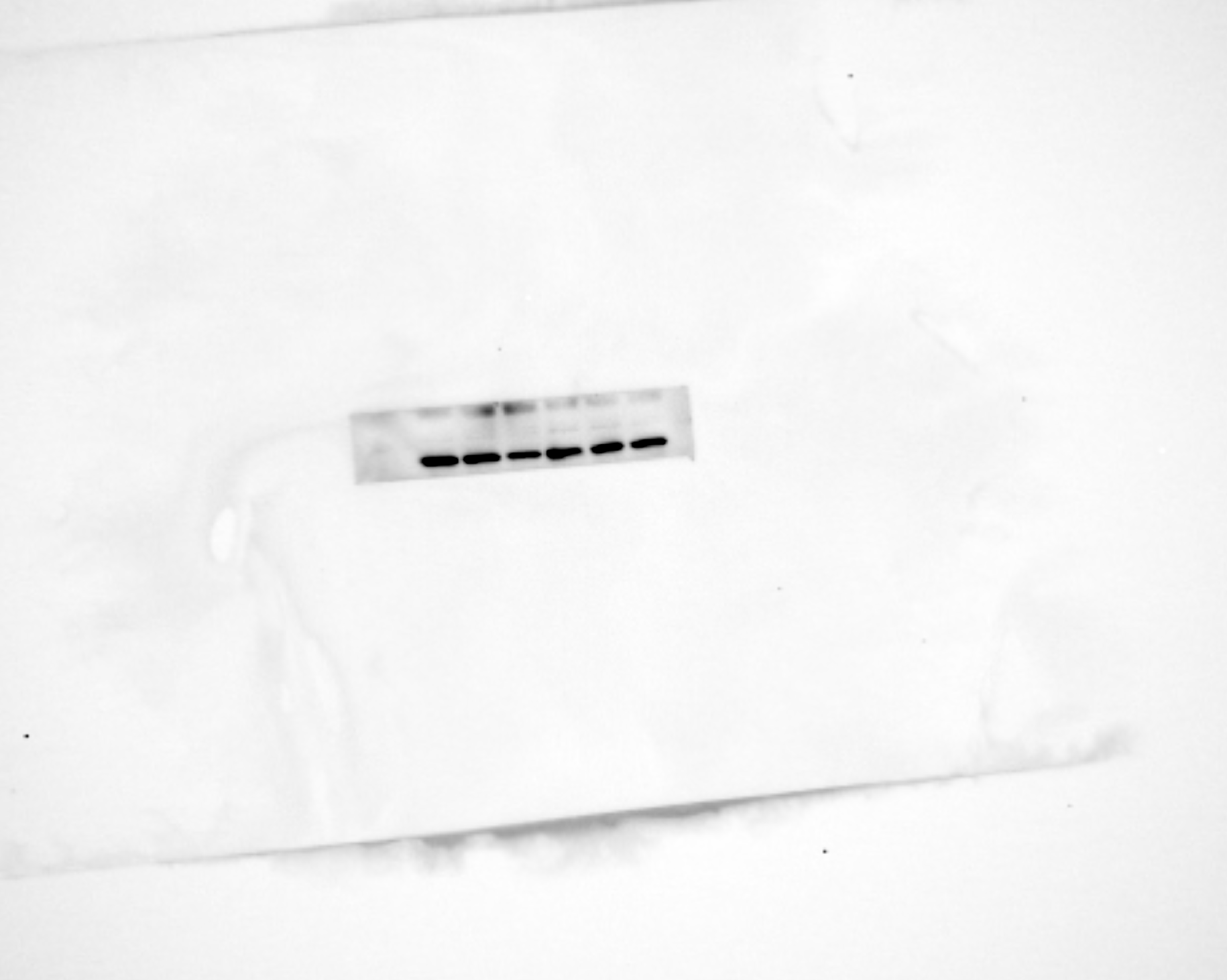

Supplement: Supplementary file 1 [file cancers-13-00807-s001.zip › Figure S4 original western blots/5E/g1-1actin_17.tif]

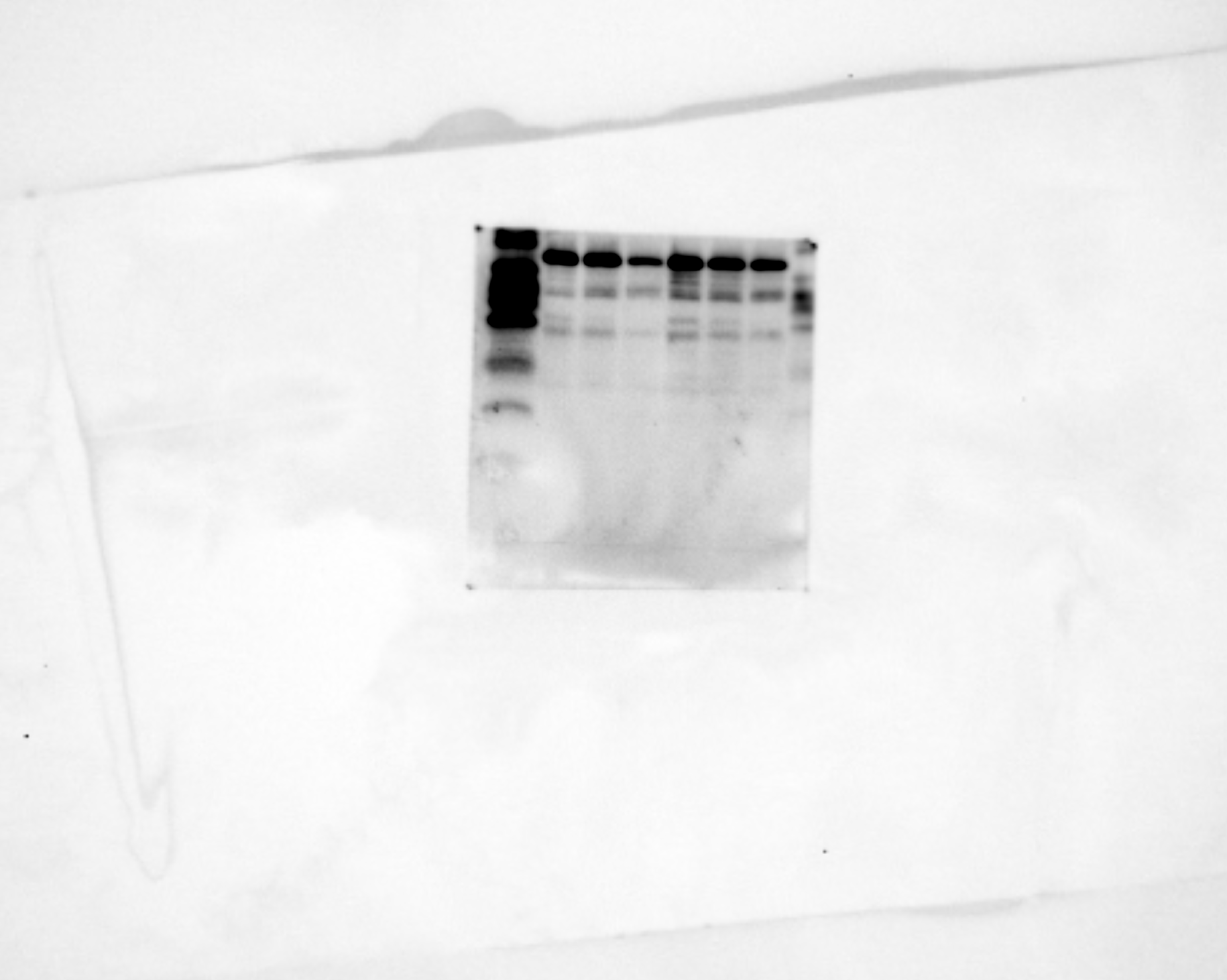

Supplement: Supplementary file 1 [file cancers-13-00807-s001.zip › Figure S4 original western blots/5E/gel1-1cas3-nostrip_15.tif]

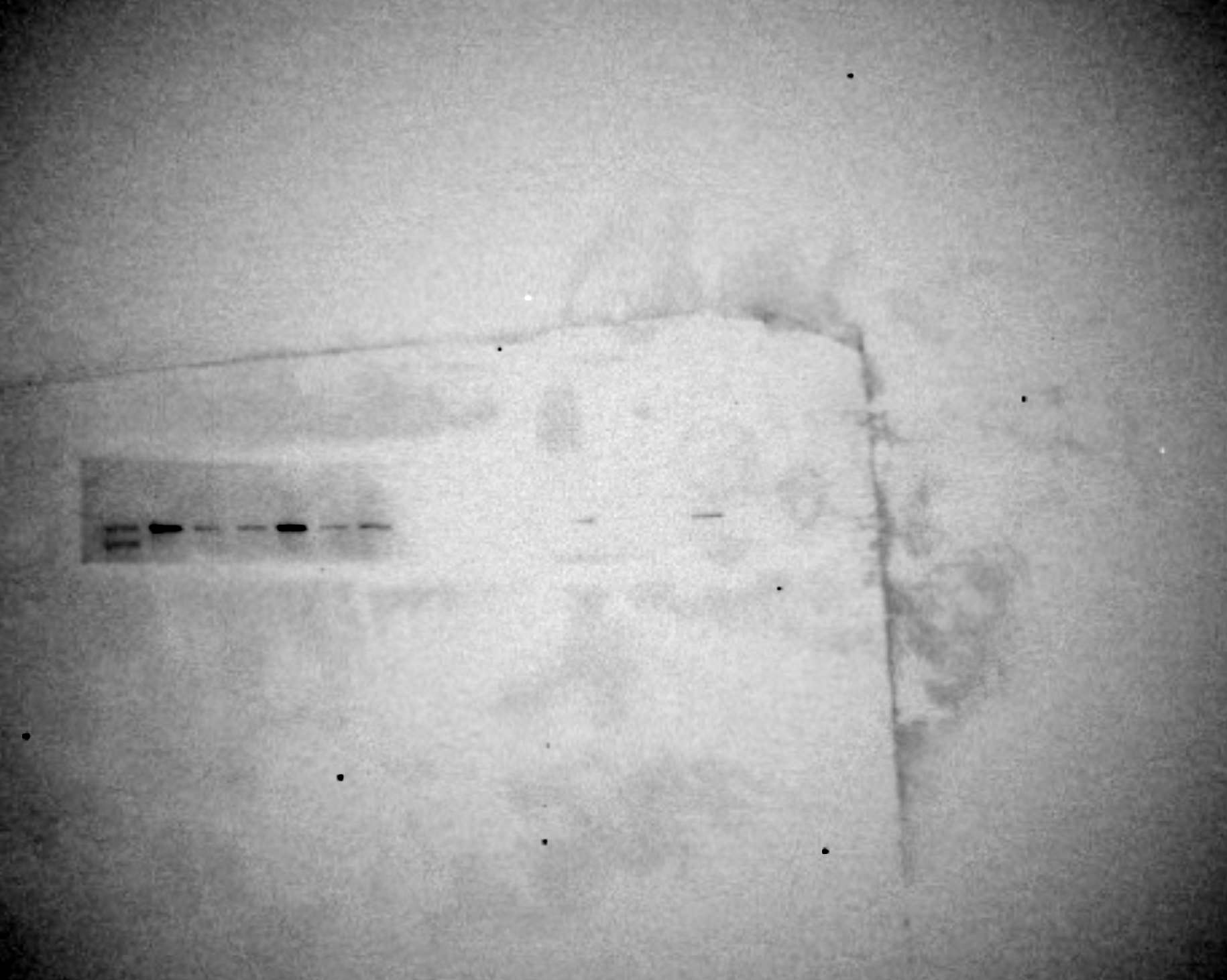

Supplement: Supplementary file 1 [file cancers-13-00807-s001.zip › Figure S4 original western blots/5E/gel1-1ecad2_26.tif]

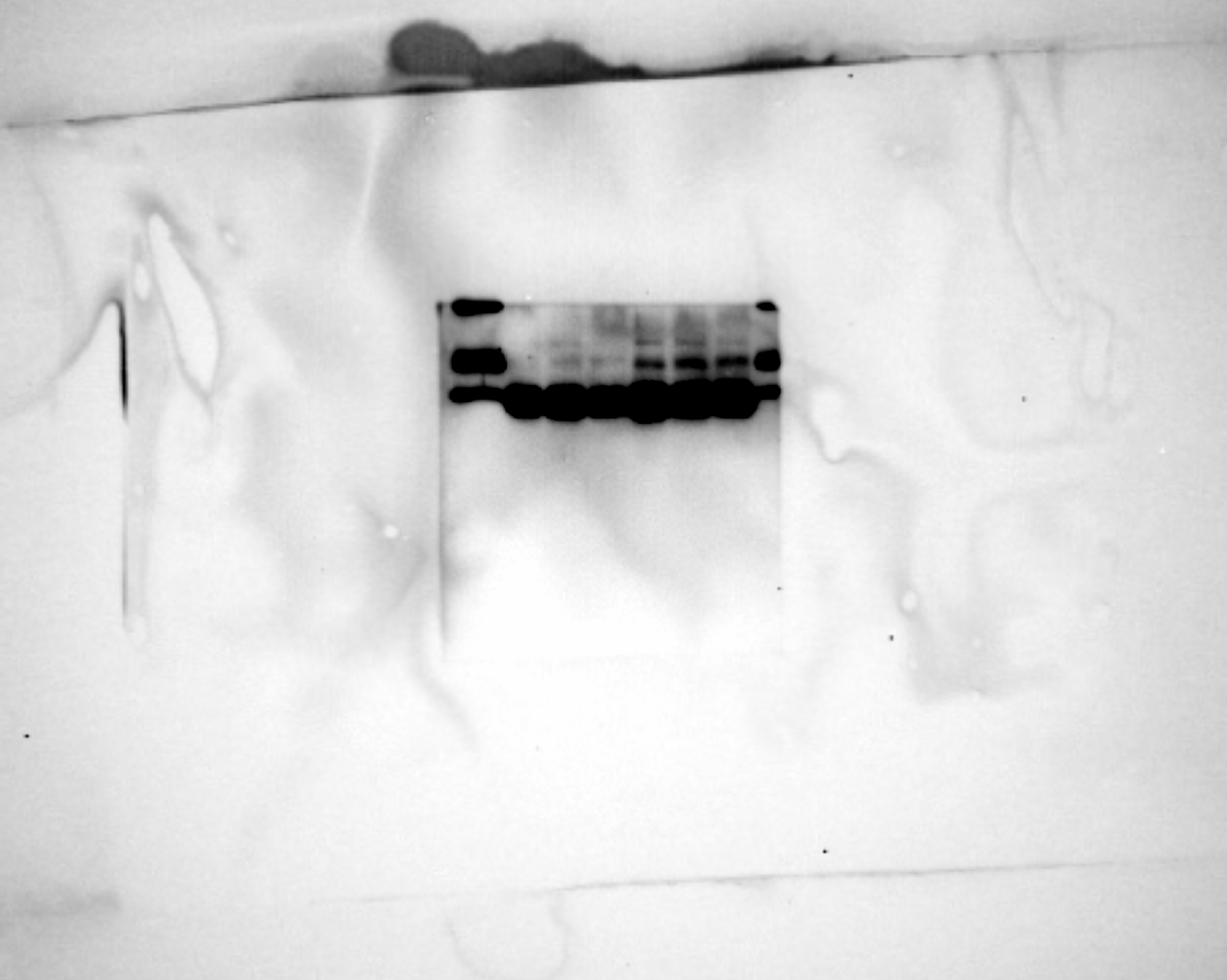

Supplement: Supplementary file 1 [file cancers-13-00807-s001.zip › Figure S4 original western blots/5E/gel1-1ras_67.tif]

## Slide 1
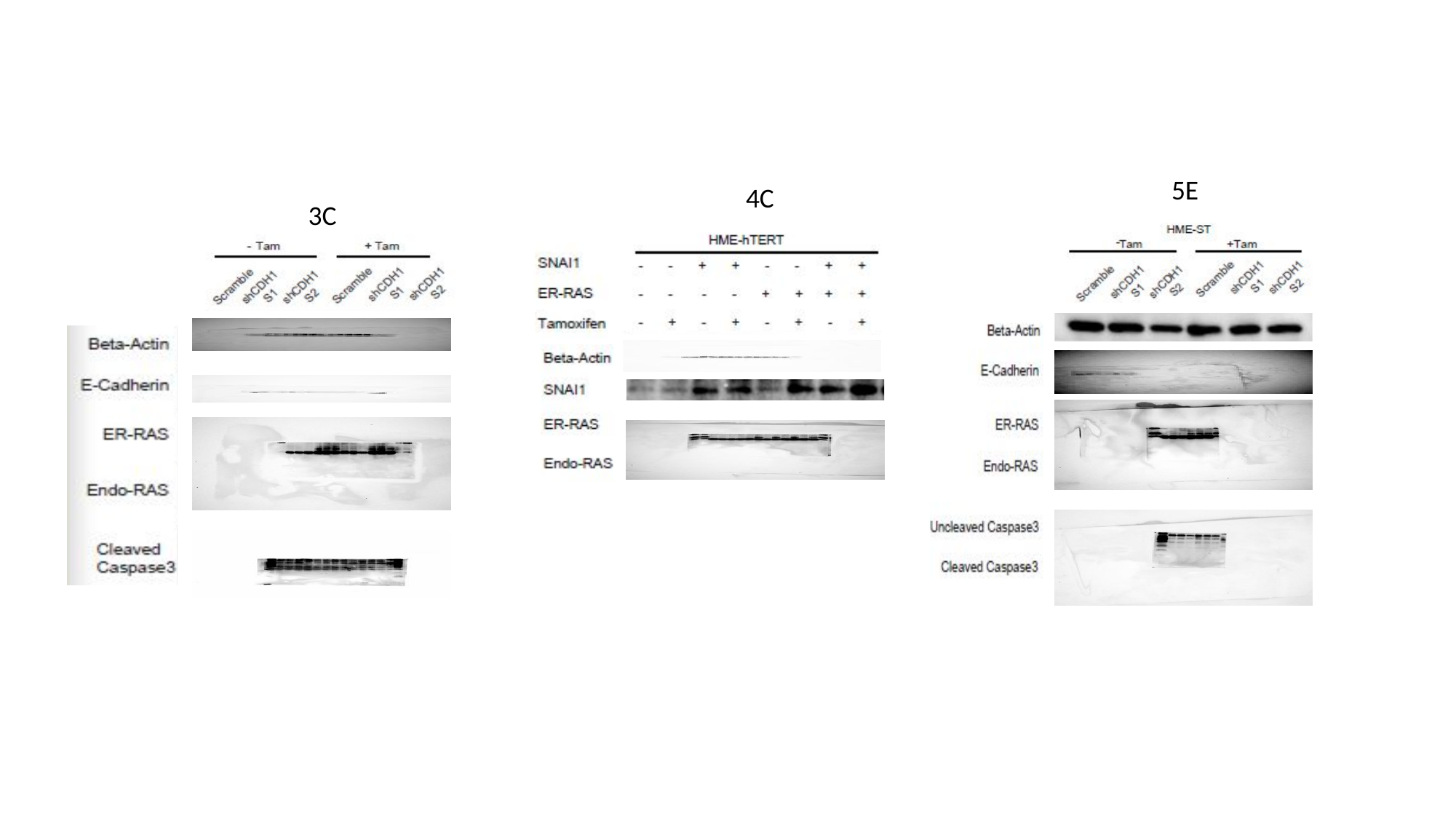

5E
4C
3C

Supplement: Supplementary file 1 [file cancers-13-00807-s001.zip › Figure S4 original western blots/compiled Figs 3C4C5E.pptx]

## Slide 1
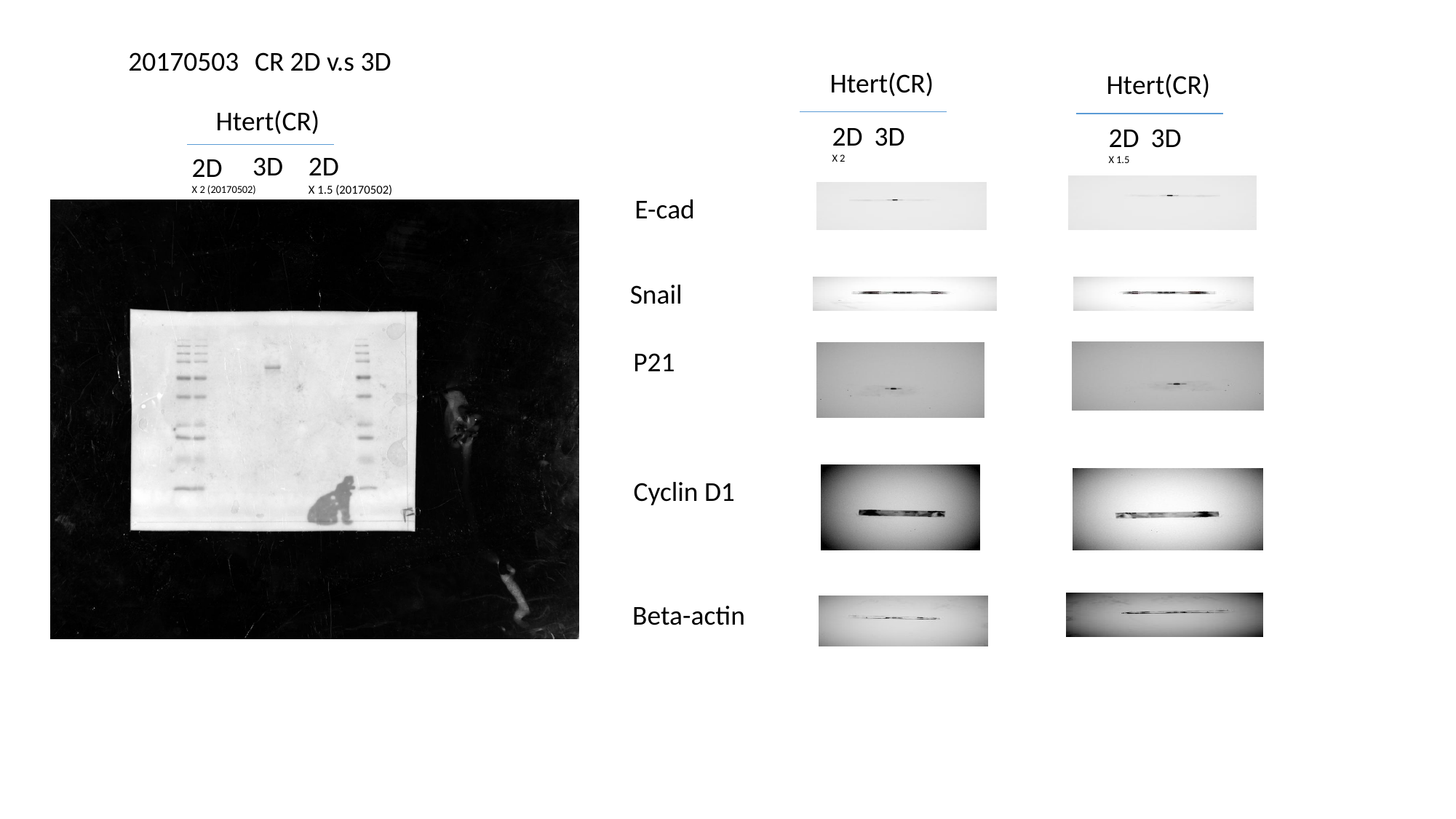

20170503
CR 2D v.s 3D
Htert(CR)
Htert(CR)
Htert(CR)
2D
X 2
3D
2D
X 1.5
3D
3D
2D
X 1.5 (20170502)
2D
X 2 (20170502)
E-cad
Snail
P21
Cyclin D1
Beta-actin

Supplement: Supplementary file 1 [file cancers-13-00807-s001.zip › Figure S4 original western blots/Fig 2B - raw data (part 1).pptx]

## Slide 1
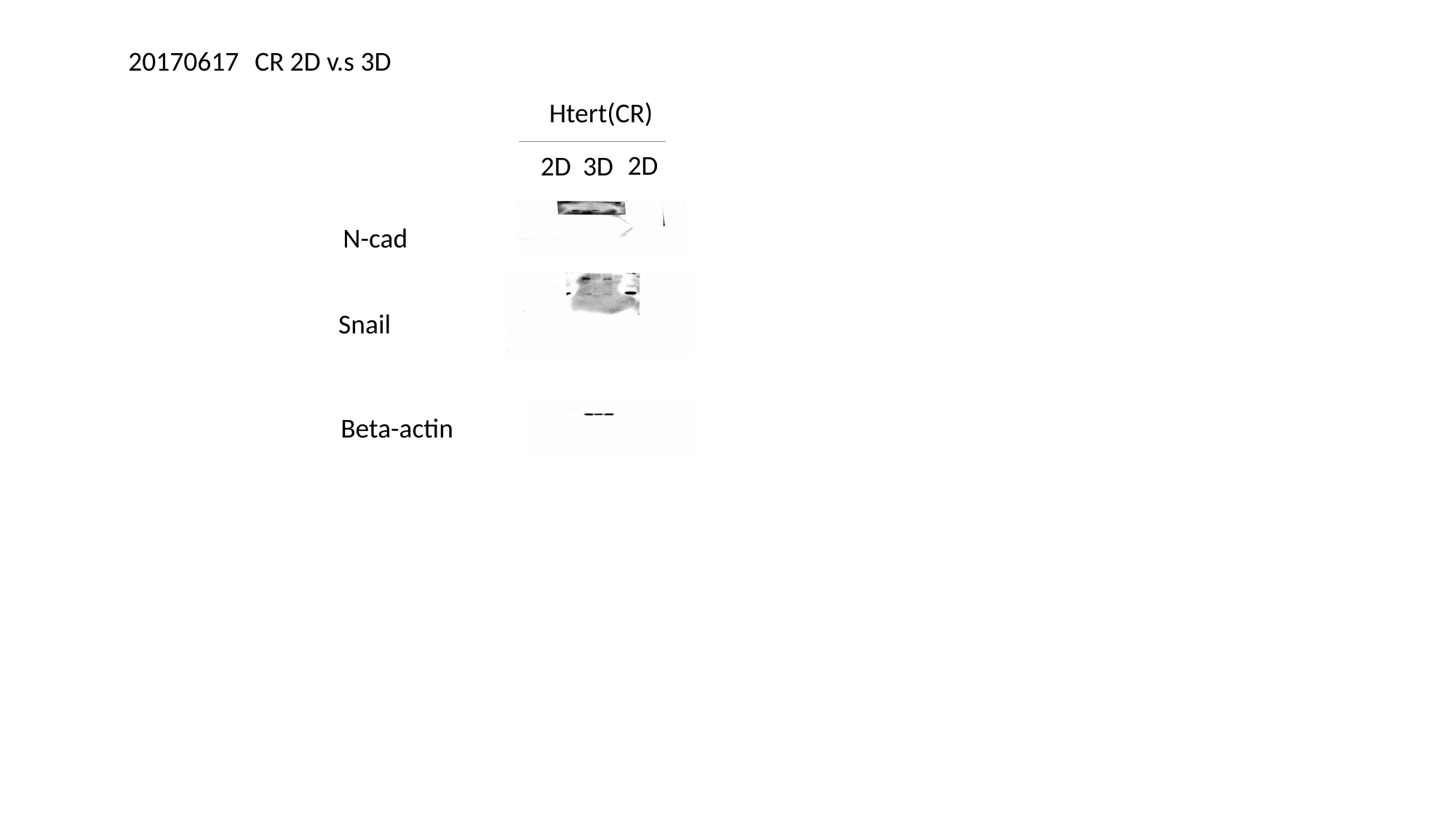

20170617
CR 2D v.s 3D
Htert(CR)
2D
2D
3D
N-cad
Snail
Beta-actin

Supplement: Supplementary file 1 [file cancers-13-00807-s001.zip › Figure S4 original western blots/Fig 2B - raw data (part 2).pptx]

## Slide 1
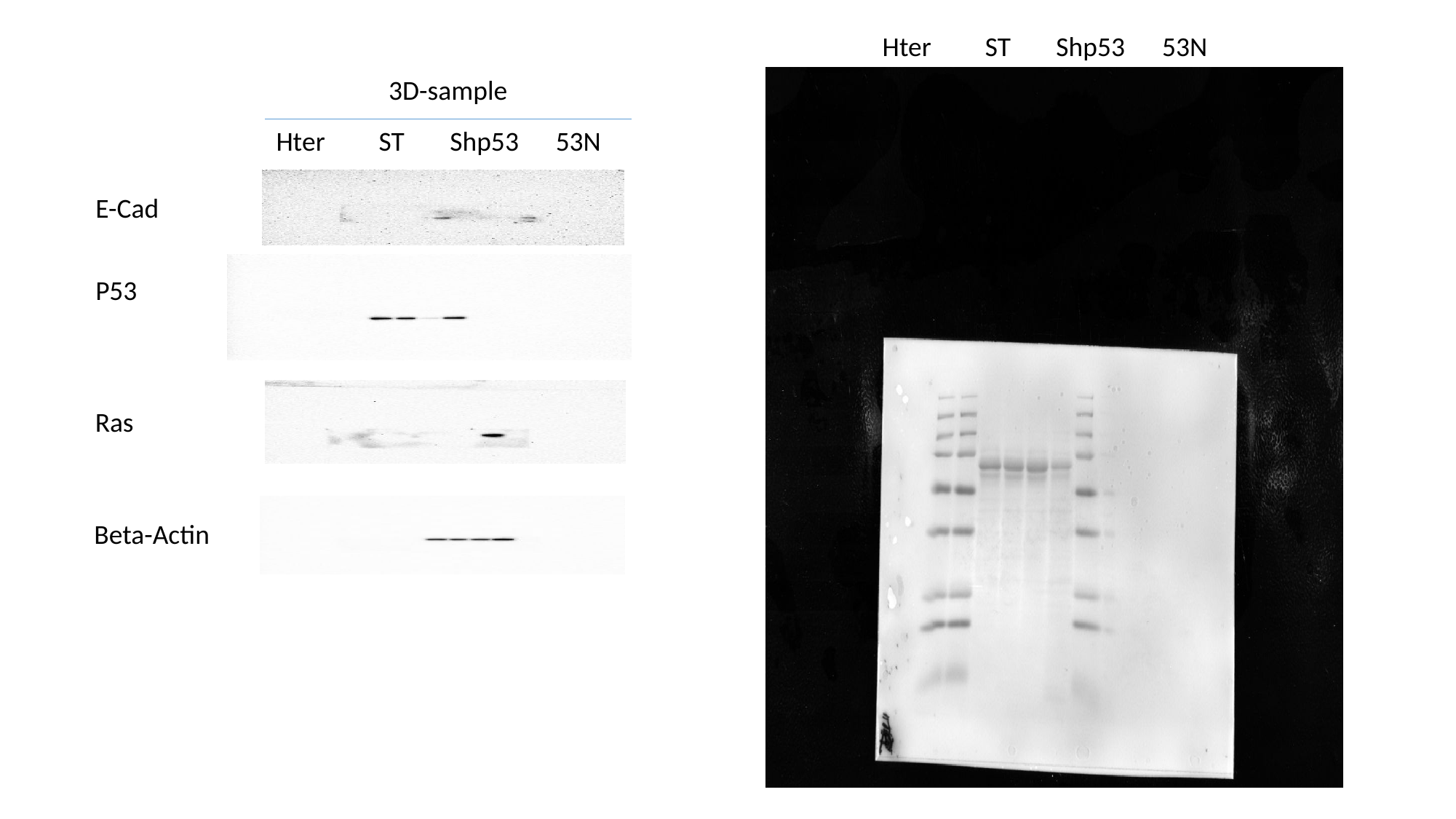

Hter
ST
Shp53
53N
3D-sample
Hter
ST
Shp53
53N
E-Cad
P53
Ras
Beta-Actin

Supplement: Supplementary file 1 [file cancers-13-00807-s001.zip › Figure S4 original western blots/Sub Fig 2E 3D - raw gel.pptx]
